# Diversity and interdomain networks of bacterial, pico-protist and nano-protist communities in a marine ranching

**DOI:** 10.3389/fmicb.2025.1620645

**Published:** 2025-07-17

**Authors:** Xinyi Zheng, Xin Guo, Xiaoqing Lin, Cheng Huang, Lingfeng Huang

**Affiliations:** ^1^Key Laboratory of the Coastal and Wetland Ecosystems, College of the Environment and Ecology, Xiamen University, Xiamen, China; ^2^State Key Laboratory of Marine Environmental Science, Fujian Key Laboratory of Marine Carbon Sequestration, College of Ocean and Earth Sciences, Xiamen University, Xiamen, China; ^3^Carbon Neutral Innovation Research Center, Xiamen University, Xiamen, China

**Keywords:** microbial network, size-fractionated protists, mariculture environment, ecology niches, seasonal dynamic

## Abstract

Microbes of diverse sizes and classifications collaborate to mediate a variety of biogeochemical processes. Although seasonal fluctuations in environmental variables generally influence microbial community dynamics, our comprehension of interdomain microbial co-occurrence patterns remains incomplete. Here, we analyzed high-throughput sequencing datasets of bacteria, pico-protists (0.8–2 μm) and nano-protists (2–20 μm), and their seasonal changes in coastal marine ranching ecosystems. Our findings revealed that, in terms of trophic groups, pico-protists predominantly comprised parasites, whereas nano-protists had a higher proportion of mixotrophs. Microbial communities shifted with seasona, mainly in response to temperature, dissolved oxygen, and salinity. Interdomain microbial networks showed the highest robustness and information transfer efficiency in autumn. This pattern was linked not only to environmental conditions but also to how specialized the protist communities became during that time. The seasonal harvesting of seaweed and stages of fish farming may have contributed to these changes. Our findings suggest that both natural seasonal cycles and mariculture activities together shape how microbial species interact, potentially affecting ecosystem stability and function.

## 1 Introduction

Bacteria and protists constitute the bulk of the marine biomass and mediated a variety of fundamental biogeochemical cycles (Pace, 1997; Landry and Schroer, 2019; Bachy et al., 2022). Advances in molecular biology have greatly enhanced our understanding of marine microbial diversity and the environmental factors shaping community composition (Kirkham et al., 2013; Sommeria-Klein et al., 2021). Bacteria are extremely abundant and diverse, and some taxa show distinct habitat preferences (Zinger et al., 2011; Flemming and Wuertz, 2019). Multigene phylogenies have divided protists into multiple supergroups, some exhibiting distinct geographic distribution patterns and variations in cell sizes (Baldauf, 2003; Massana, 2011; Sommeria-Klein et al., 2021). Based on cell size, protists subdivide into pico-protists (< 2 μm) and nano-protists (2–20 μm) (Sieburth et al., 1978; Caron et al., 2017). Pico-protists were considered to be predominantly phototrophic, with heterotrophic taxa accounting for only 20–30% of the abundance (Jürgens and Massana, 2008). Nano-protists have diverse trophic modes and are key links in the food web, influencing the marine carbon cycle (Worden et al., 2015). Interactions such as predation, competition, parasitism, and symbiosis occur among microorganisms across different sizes and taxonomic groups (Azam and Malfatti, 2007; Worden et al., 2015; Caron et al., 2017). Understanding and characterizing their diversity and co-occurrence patterns is essential for deciphering how these intricate microbial communities respond to environmental changes and contribute to broader ecosystem dynamics (Edwards and Richardson, 2004; Azam and Malfatti, 2007; Cavicchioli et al., 2019).

Species co-occurrence is primarily shaped by biological interactions, environmental filtering, and dispersal constraints (Berry and Widder, 2014). Ecological networks are the fundamental tools for exploring microbial co-occurrence patterns in ecosystems, which can identify potential interactions among organisms, detect keystone species, and pinpoint core components in ecosystems (Fuhrman, 2009; Fuhrman et al., 2015; Röttjers and Faust, 2018). Previous studies have revealed distinct differences between bacterial and eukaryotic network structures within the same environment, with some exhibiting even opposing responses to identical environmental changes (Zhu W. et al., 2022; Chen et al., 2023). Thus, incorporating interdomain microbes and analyzing networks across multiple size fractions is vital for accurately assessing environmental influences on microbial co-occurrence patterns (Xue et al., 2022; Shekarriz et al., 2023). Currently, most network analysis tools were based on correlation methods, and study sample sizes were typically in the tens to hundreds, which might lead to spurious results (Friedman and Alm, 2012; Kurtz et al., 2015). The SPIEC-EASI (SParse InversE Covariance Estimation for Ecological Association Inference) algorithm, designed for amplicon sequencing data, employs sparse neighbor and inverse covariance selection techniques to improve the reliability of microbial ecological network inference and offer a more precise understanding of microbial community co-occurrence patterns (Kurtz et al., 2015).

Environmental filtering shapes microbial communities through both abiotic factors—such as temperature, salinity, and nutrient availability—and biotic factors, including the diversity and abundance of predators (such as nano-protists) and prey (such as bacteria and pico-protists) (Moran, 2015; García-Comas et al., 2016; Yang et al., 2018; Sommeria-Klein et al., 2021; Guo et al., 2023). Microorganisms respond differently to environmental changes and are therefore classified as either habitat generalists, which adapt to flexibly to environmental fluctuations, or habitat specialists, which occupy narrow niches (Kneitel and Chase, 2004; Muller, 2019). Niche-based assessment contributes to disentangling microbial community co-occurrence patterns and better predicts ecosystem fate (Muller, 2019). Recent findings indicated that microbial generalists act as keystone species supporting and connecting the anthospheric microbiome (Kim et al., 2025). Conversely, studies on sediments and bays suggested that specialists play a greater role in maintaining the stability of microbial co-occurrence networks (Mo et al., 2021; Yan et al., 2022). A study on co-occurrence networks was shown that generalists and specialists had similar degrees of connections, indicating that both groups were important for network robustness (Xu et al., 2022). Nevertheless, it remains unclear whether generalists or specialists contribute more to the stability of co-occurrence patterns.

To decipher the co-occurrence pattern of multiple sized-fractionated microbial communities, we conducted a study in Sansha Bay, Fujian Province, China. Sansha Bay is a semi-enclosed, bay-type marine ranching with a about 2.9-km outlet, featuring a polytrophic mariculture system that includes cage farming and algae cultivation. The culture pattern showed a distinct seasonality. Cage culture was mainly for large yellow croakers (*Larimichthys crocea*), with juveniles generally reared in nets in the spring and autumn of annually. Algae culture mainly consists of kelp (*Saccharina japonica*), cultured from December to May, and seaweed (*Gracilaria lemaneiformis*), cultured year-round. The waters are often in eutrophic conditions as a consequence of mariculture bait inputs. To determine how multiple sized-fractionated microbial communities respond to seasonal dynamics and mariculture activities, we investigated the diversity and structure of bacterial, pico-protist and nano-protist communities and quantified the contribution of environmental factors to microbial co-occurrence network structure. The aim was to: (i) identify functional differences among microbial communities across distinct size fractions; (ii) examine seasonal variations in habitat specialists and generalists; and (iii) determine how environmental variables and mariculture activities shape microbial co-occurrence patterns. We hypothesized that microbial ecological functions significantly differ across various size-fractionated groups, and that specialists play a pivotal role in shaping interdomain microbial networks.

## 2 Materials and methods

### 2.1 Sample collection

**Figure 1:**
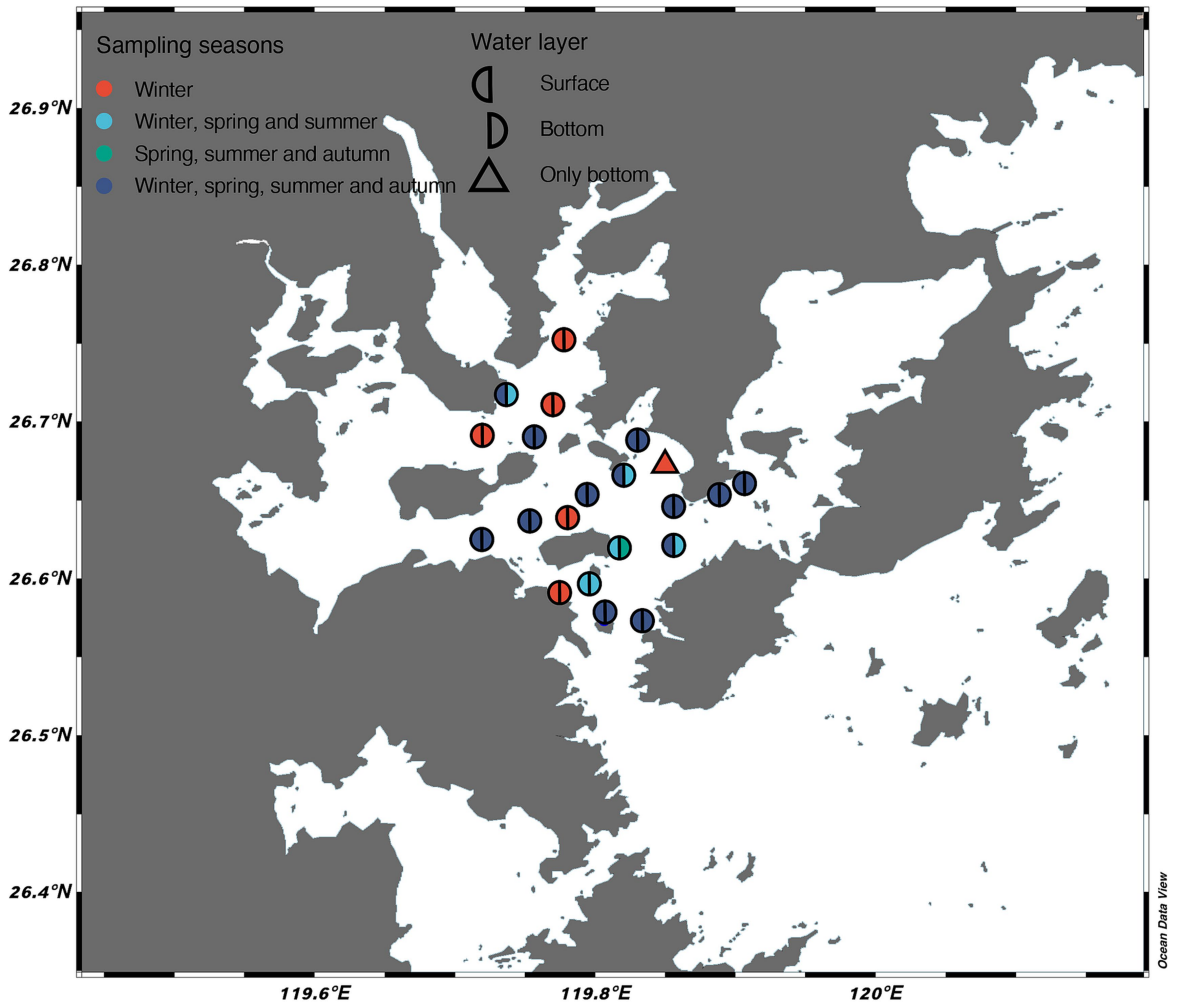
Location of sampling stations in Sansha Bay, Fujian province (China). The colors and symbols represent sampling seasons and water layers.

Samples (*n* = 124) were collected from a near-enclosed mariculture bay at the water layer of the surface (0.5 m) and bottom (2–55.5 m) from the Sansha Bay, Fujian Province during four cruises (January, April, July, and October 2019, i.e., winter, spring, summer, and autumn) (Figure 1). Water samples were prefiltered through a 200 μm pore mesh. A portion of water samples were directly filtered onto a 0.22 μm filter membrane (47 mm diameter; Millipore, United States) to obtain bacterial samples. Another portion of water samples were prefiltered through a 20 μm pore mesh and then sequentially filtered to 2 μm and 0.8 μm filter membranes (47 mm diameter; Millipore, United States) to obtain the samples of pico-protists (0.8–2 μm) and nano-protists (2–20 μm). These filters were stored at −80°C until DNA extraction.

### 2.2 Measurement of environmental factors

Water temperature, salinity and depth were measured *in situ* with CTD (AML Base X, Canada). Dissolved oxygen (DO) were measured in situ with WTW (Multi3630 IDS, German). Nitrate (NO_3_-N), nitrite (NO_2_-N), ammonia (NH_4_-N), phosphate (PO_4_-P), silicic (Dsi), chlorophyll *a* (Chl*a*), as well as dissolved total nitrogen (TN), total phosphorus (TP), were measured following the standardized method (Xie et al., 2020). Dissolved inorganic nitrogen (DIN) content was calculated by the sum of NO_3_-N, NO_2_-N, and NH_4_-N.

Water samples pre-filtered at 20 μm were fixed with glutaraldehyde at a final concentration of 0.1% (V/V) and the abundance of heterotrophic bacteria (HB), *synechococcus* (Syn) and photosynthetic picoeukaryotes (PPE) were run on a FACSAria flow cytometer (Becton Dickinson, United States) equipped with laser emitting at 488 nm (Marie et al., 1997, 2000). To measure the abundance of HNF (heterotrophic nanoflagellates) and PNF (pigmented nanoflagellates), water samples pre-filtered at 20 μm were fixed with glutaraldehyde at a final concentration of 0.5% (V/V), then stained with 4′6-diamidino-2-phenylindole (DAPI), and filtered at low pressure (<100 mm Hg) onto 0.8 μm pore-size black nuclear pore filters (25 mm diameter; Millipore, United States). Filters were mounted to glass slides and stored at −20°C in the dark until observed by epifluorescence microscope (Leica Microsystems, Germany). Following Guo et al. (2020) methods, at least 50 randomly selected fields of view were examined for each sample.

Conversion of enumerated microbial abundance to carbon-containing biomass with reference to survey data in the adjacent sea area. The abundance of HB and Syn was converted to the biomass using a factor of 20 fg C cell^−1^ and 178 fg C cell^−1^, respectively (Lee and Fuhrman, 1987; Charpy and Blanchot, 1998). The abundance of PPE was converted to biomass using a factor of 1,500 fg C cell^−1^ (Zubkov et al., 1998). The abundance of HNF and PNF was converted to biomass using a factor of 4,700 fg C cell^−1^ (Chen et al., 2009).

### 2.3 DNA extraction, PCR amplification, and high-throughput sequencing

For protists, DNA was extracted using DNeasy PowerWater kit (Qiagen, United States), the V4 region of the 18S rRNA gene was amplified with primers TAReuk454FWD1 (5′-CCAGCA(G/C)C(C/T)GCGGTAATTCC-3′) and TAReukREV3 (5′-ACTTTCGTTCTTGAT(C/T)(A/G)A-3′) (Stoeck et al., 2010). A quintuple repetition of each sample was amplified as follows: 95°C for 5 min, 10 cycles of 94°C for 30 s, 47°C for 45 s, and 72°C for 1 min, 25 cycles of 94°C for 30 s, 47°C for 45 s, and 72°C for 2 min, and a final extension at 72°C for 2 min. Forward and reverse primers were tagged with 2 bp links and 8 bp barcodes to allow the pooling of multiple samples in one run of sequencing and later differentiation of different samples (Kozich et al., 2013). The PCR products were purified using Agarose gel DNA Recovery Kit (Bioteke, China) and quantified by Nano-200 (Allsheng, China). Samples belonging to two size-fractionated were, respectively, mixed in equimolar concentrations to construct two individual amplicon libraries for sequencing using Illumina Hiseq 2500 platforms with PE250 strategy (Illumina, United States) according to standard protocols. The raw sequence data have been deposited in the NCBI Sequence Read Archive under the BioProject accession number PRJNA1252866.

For bacteria, 16S rRNA gene sequences were obtained from the BioProject number PRJNA747131 and the Accession number SRP328863 (Zhu J. et al., 2022). Briefly, DNA was extracted using the FastDNA spin kit (MP Biomedicals, USA), the V3-V4 region of the 16S rRNA gene was amplified with primers 341F (5′-CCTAYGGGRBGCASCAG-3′) and 806R (5′-GGACTACNVGGGTWTCTAAT-3′) (Liu et al., 2019). The triplicate PCR products were purified using GeneJET gel purification kit (Thermo Scientific, United States) and quantified by Qubit 2.0 fluorescence quantifier (Thermo Fisher Scientific, Waltham, United States). PCR products were mixed in equal amounts and sequenced on THE Illumina HiSeq platform (Illumina, United States).

### 2.4 Sequence analysis

Sequencing data processing was performed on Mothur v.1.47.0 following MiSeq standard operating procedure^1^ with the steps of sequencing data quality control, amplicon sequence variants (ASV) clustering, and species classification. Specifically, read pairs were aligned, and the tags and primers of reads were removed. To reduce sequencing and PCR error, reads with only one or two sequences were removed. Chimeras were detected using the UCHIME algorithm (Edgar et al., 2011), and if there were flagged chimeras, they were removed from all samples. The remaining high-quality reads with suitable lengths were clustered to distinguish ASVs. The SILVA nr v.138 database (Quast et al., 2012) and PR^2^ protist v4.14 database (Guillou et al., 2012) were used for bacterial and protist taxonomic assignment, respectively. To avoid distortion of the relative abundance of DNA sequences of microbes, non-bacterial or non-protistan sequences (e.g., “unknown,” Archaea, Nucleomorphs, Fungi, Streptophyta, and Metazoa) were removed. To minimize the occurrence of ASVs at both size-fractionated due to cell dislodging or filter clogging, ASVs were further filtered with reference to Deutschmann et al. (2023). For each ASV that appeared at both size-fractionated, the ratio of its relative abundance at the nano-and pico-protist communities was calculated, and when the ratio exceeded 2, the abundance of pico-protists was removed. When the ratio was lower than 0.5, the abundance of nano-protists was removed. Lastly, 124 valid samples were obtained, including 40 winter samples, 30 each of spring and summer samples, and 24 autumn samples. A total of 9,025 bacteria ASVs, 12,274 pico-protist ASVs, and 12,908 nano-protist ASVs were retained.

Referring to previous literature reports (Adl et al., 2012; de Vargas et al., 2015b), the functional groups of pico-and nano-protist ASVs were annotated into photoautotroph, symbiont, parasites, heterotroph, and mixotroph. Here, mixotrophs refer to photosynthetic species that also ingest food by phagocytosis or osmotrophy, while symbiont refers to heterotrophic species that retain prey plastids or symbionts (Adl et al., 2019) (Supplementary Appendix A).

### 2.5 Statistical analysis

All statistical analyses were performed in R v.4.2.1 software (R Core Team, 2019), and visualized using the “ggplot2” R package (Wickham, 2016). The α-diversity indices, including Shannon, Chao1, and Pielou’s evenness, were calculated using the “vegan” R package (Oksanen et al., 2022). Differences in α-diversity among seasons were analyzed with multiple comparisons (LSD test), using the “agricolae” R package (Mendiburu, 2010).

Prior to multivariate statistical analyses, the ASV tables were Hellinger-transformed and the environmental variables were standardized to zero mean and unit variance by “vegan” R package (Oksanen et al., 2022). The β-diversity was calculated on the Bray-Curtis distance metric and visualized with nonmetric multidimensional scaling (NMDS). The significant differences in microbial communities among water layers were tested by permutation multivariate analysis (PERMANOVA) of variance using the Bray-Curtis distance metric. To explore the correlations between environmental factors and their effects on microbial communities, Spearman correlation analysis and partial Mantel test were performed using the “microeco” R package (Liu et al., 2020). For the partial Mantel test, the distance matrix of microbial communities was calculated using the Bray-Curtis method. Additionally, in order to reveal the biotic interactions between size groups, the affecting biotic factors for each size group did not contain the abundance data of the same size of the organisms. To further identify characteristic taxa, the linear discriminant analysis (LDA) effect size (LEfSe) was used to screen for classes that differed significantly between seasons (LDA > 4.0) and analyze.

To assess the environmental adaptability of microbial communities, the community-level niche breadth was calculated, which represents the average ecological tolerance of all taxa in a community. A higher niche breadth indicates a predominance of generalist species, while a lower value reflects a dominance of specialists with narrower ecological preferences (Pandit et al., 2009). This index was calculated as the mean of Levins’ niche breadth values for all taxa within a community (Pandit et al., 2009; Wu et al., 2018). Levins’ niche breadth index (B) was calculated following the description of Pandit et al. (2009) using the “spaa” R package (Zhang, 2016): $B_{j}=1/\sum_{i=1}^{N}P_{\mathrm{ij}}^{2}$ , where *B_j_* represents the habitat niche breadth of ASV *j* in a metacommunity; *N* is the total number of communities in the metacommunity; and *P_ij_* is the proportion of ASV *j* in resource state *i*, i.e., the abundance of ASV *j* in community *i* divided by the abundance of ASV *j* in the metacommunity. The *B* value ranges from [1, *N*] and the higher value indicates that the ASV *j* is widely and evenly distributed in the metacommunity. Community-level niche breadth was calculated for all seasons, and metacommunities were defined as the set of communities in each season. Differences in community-level niche breadth among communities were analyzed with the LSD test.

### 2.6 Ecological networks construction

All samples were used for network inference (*n* = 124). To focus on widespread interactions among community members and their relative influence on network properties, ASVs shared by all seasons, presented in more than 1/3 of samples and with an average relative abundance greater than 0.001 were retained (*n*_Bac_ = 42, *n*_Pico-protist_ = 85, *n*_Nano-protist_ = 100). Networks were generated using the “SpiecEasi” R package relying on sparse neighborhood and inverse covariance selection algorithms (Kurtz et al., 2015). Interdomain and intradomain networks were constructed independently using the sparse and low rank (SLR) method by setting parameters (nlambda = 20, minimum lambda ratio = 0.005, pulsar threshold = 0.05, number of representatives = 20) (Shekarriz et al., 2023).

Network topology parameters (i.e., degree, absolute, and betweenness centrality) were calculated using the “igraph” R package, and network modularization and visualization were performed using Gephi software. Referring to the code provided by Shekarriz et al. (2023), we analyzed the efficiency and random attack robustness of networks, which were proposed by Latora and Marchiori (2001) and Iyer et al. (2013), respectively. Normalized robustness (*R*) of a network was calculated by running iteratively for 10,000 times, using the formula $R=\frac{1}{N}\sum_{i=1}^{N}\sigma (\frac{1}{N})$ , where *N* is the initial size of the network, *σ* is the relative size of the largest network component after node removal, and *i* is the number of vertex or vertices removed from the network. The vulnerability (*V*) of a network is related to the robustness calculated as V=0.5−R . Multiple comparisons of the network properties were conducted using the LSD test. Keystone species were identified as nodes with degree and betweenness centrality measures with values in the top 20 percentiles (Roume et al., 2015).

### 2.7 Partial least squares path model analysis

To further reveal the effects of environmental variables on microbial communities and microbial food web structure, a partial least squares path model (PLS-PM) was modeled using the “plspm” R package (Sanchez et al., 2024). The model included microbial community diversity, community-level niche breadth, and network information transfer efficiency. Network information transfer efficiency index reflects how effectively signals or interactions propagate through the microbial network, and can serve as a proxy for the functional coordination and resilience of the microbial system. Following García-Comas et al. (2016) and Yang et al. (2018), we also included the predator/prey biomass ratio (PPBR) as a proxy for the actual trophic transfer efficiency between predator and prey to assess its ecological relationship to mathematically inferred network connectivity and flow. To improve the reliability of the model, the PPBR was log10 transformed, run using 1,000 bootstraps, and variables with loadings < 0.7 were removed. Based on the path coefficients, the direct and indirect effects of other potential variables on network robustness and information transfer efficiency were calculated. The performance of the model was evaluated using the Goodness-of-Fit (*gof*) measure. The final PLS-PM model included seven variables: water property (temperature, salinity, and DO), water nutrition (TN and TP), microbial community diversity (only pico-protist and nano-protist), community-level niche breadth (all three groups), PPBR (the biomass ratio of HNF/bacteria and HNF/PPE), network robustness, and network information transfer efficiency.

## 3 Results

### 3.1 Environmental condition of Sansha Bay

Environmental conditions varied more in temporal than in spatial variations, with little difference in environmental parameters between two depths in the same season (Supplementary Figure S1). The environment was nitrogen nutrient-rich, with average PO_4_-P, DIN, and nitrogen-to-phosphorus ratios (TN: TP) reaching eutrophic levels in autumn and winter, and middling nitrogen-rich nutrient levels in spring and summer (Supplementary Table S1). Temperatures, DO, PO_4_-P, and NH_4_-N in Sansha Bay were distinct in all seasons (*p* < 0.05, Supplementary Figure S1). Microbial biomass also fluctuated seasonally (*p* < 0.05). In winter, the abundance of pico-photosynthetic microorganisms (PPE and Syn) was at a low level, while the abundance of PNF reached its peak throughout the year. The abundance of HB and HNF showed a similar trend of being higher in winter and summer, and lower in spring and autumn (Supplementary Figure S1).

### 3.2 Diversity and structure of bacterial and protist communities

**Figure 2:**
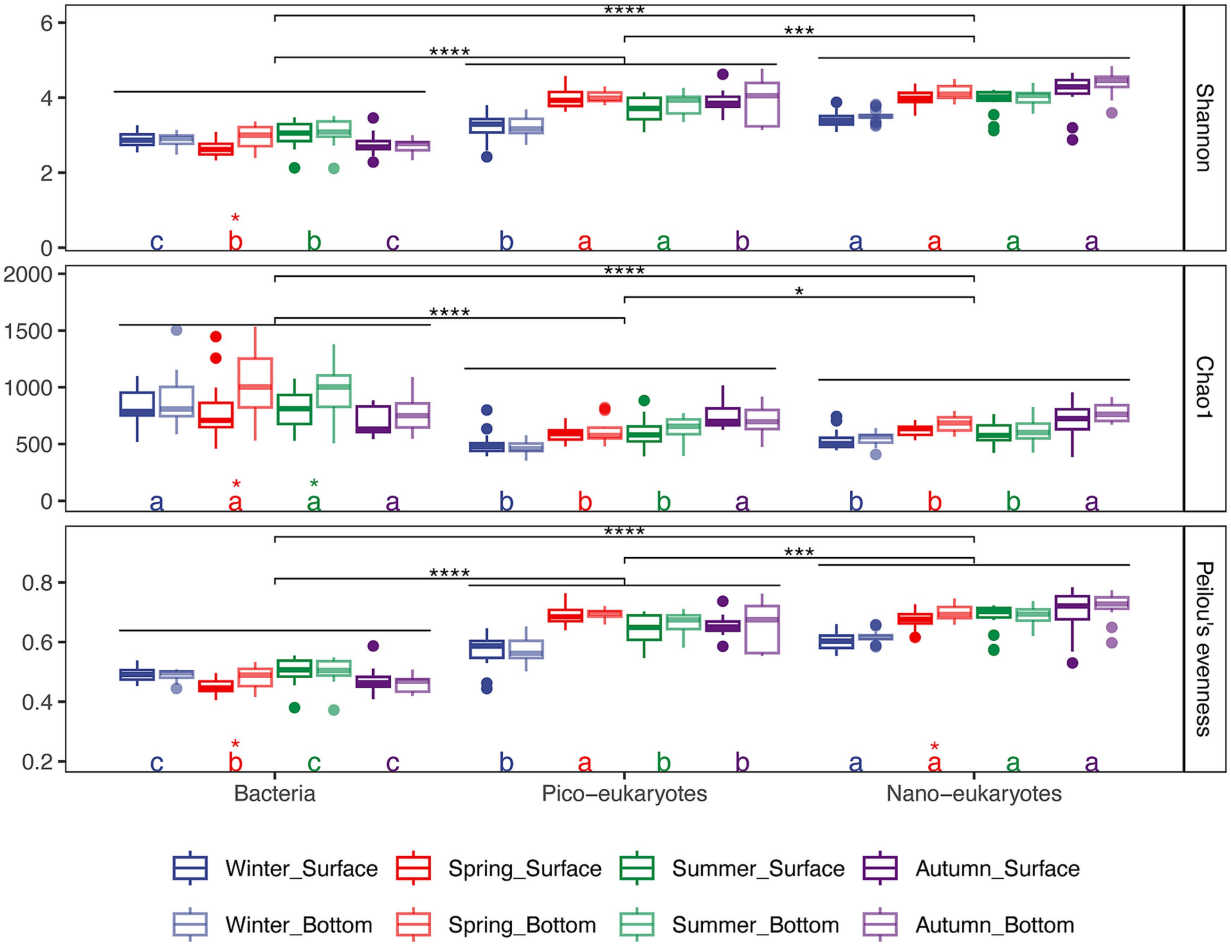
Comparison of the α diversity of bacterial, pico-protist, and nano-protist communities in four seasons. Different letters indicate statistically significant differences (*p* < 0.05) based on LSD tests; groups sharing the same letter are not significantly different. Black asterisks represent the significance of differences between groups of bacterial, pico-, and nano-protist communities. Colored asterisks represent the significance of differences between water layers in the same season for a given taxon. Significance level: *p < 0.05; **p < 0.01; ***p < 0.001; ****p < 0.0001.

Departing from previous investigations by Ma et al. (2021) and Zhu J. et al. (2022) that focused on bacteria and protists (0.22–200 μm) diversity, here we delved into the diversity of pico and nano size-fractionated protists. We provided a detailed description of changes in community composition and interdomain networks, aiming to elucidate the connections between protist and bacterial communities. The diversity indices of the three groups were significantly different (*p* < 0.05, Figure 2). The Shannon index and Peilou’s evenness index of the nano-protist community were the highest, followed by the pico-protists and bacteria, while the chao1 index was the opposite. The α-diversity indices of bacterial and protist communities showed greater seasonal than depth variation (Figure 2). The pico-protist and nano-protist community diversity indices were comparable, both fluctuated upward over time and appeared to have complementary seasonal fluctuations to the bacteria (Supplementary Figure S2).

**Figure 3:**
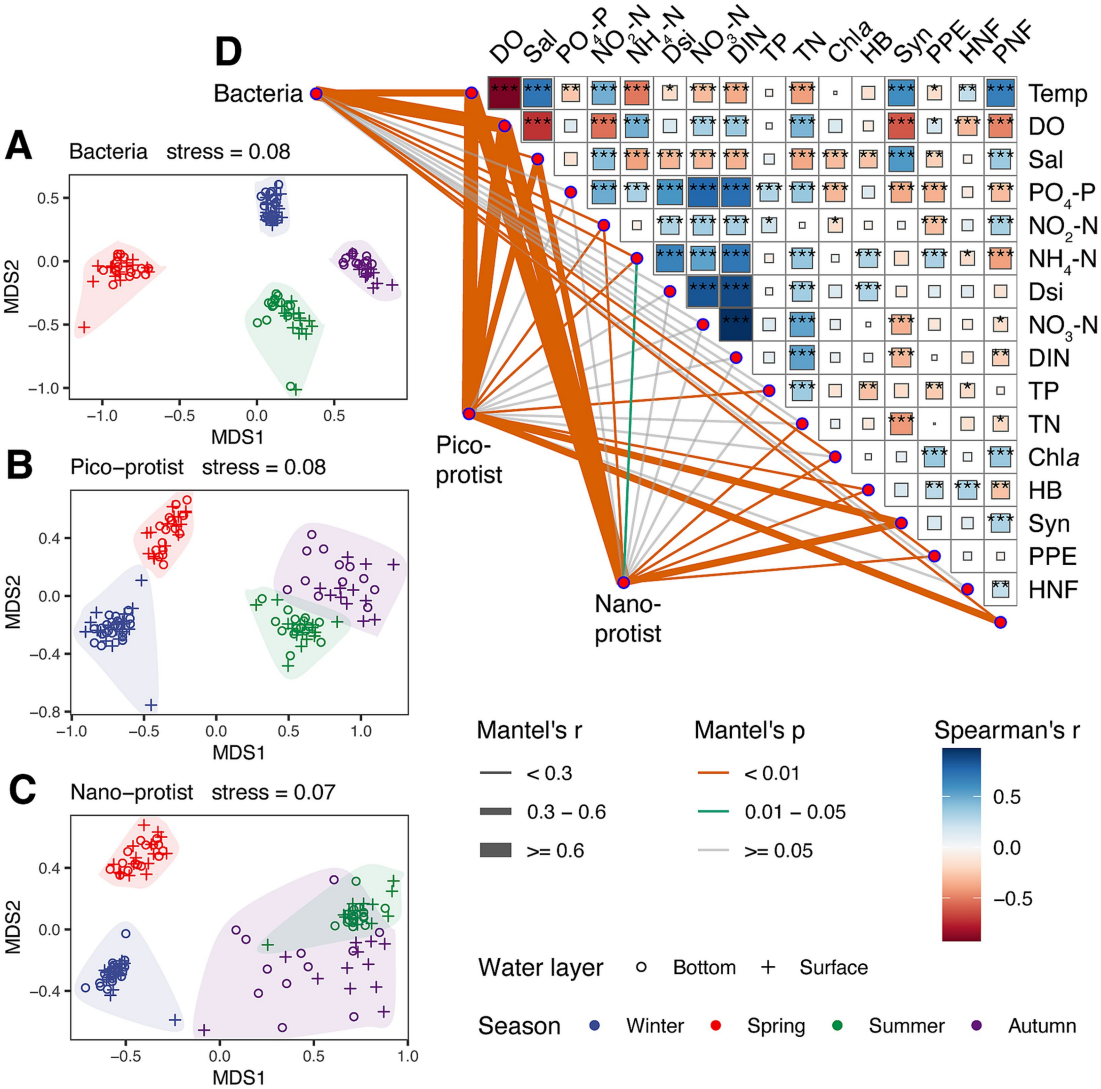
Non-metric multidimensional scaling analysis (NMDS), pairwise comparisons of environmental factors, and mantel tests for the correlations between the bacterial, pico-protist and nano-protist community and each environmental factor. **(A–C)** NMDS analysis of bacteria, pico-protist and nano-protist communities of all stations. The color of the scattered dots represents the season of the station, and the shape of the scattered dots represents the water layer of the station. **(D)** Interactions across environmental factors and their relationship with bacteria, pico-protist, and nano-protist communities. The thickness of the line indicates the mantel correlation between the environmental factor and the microbial communities, and the color of the line indicates the significance level of the mantel correlation. The intensity of the filled color of the squares indicates the correlation between the environmental factors, ranging from red (negative interaction), and white to blue (positive interaction). Asterisks indicate significance level: *p < 0.05; **p < 0.01; ***p < 0.001. Temp, Temperature; DO, dissolved oxygen; Sal, salinity; PO_4_-P, phosphate; NO_2_-N, nitrite; NH_4_-N, ammonia; Dsi, silicic; NO_3_-N, nitrate; DIN, dissolved inorganic nitrogen; TP, total phosphorus; TN, total nitrogen; Chl*a*, chlorophyll *a*; HB, heterotrophic bacteria; Syn, *Synechococcus*; PPE, photosynthetic picoeukaryotes; HNF, heterotrophic nanoflagellates; PNF, pigmented nanoflagellates.

The community structure of bacteria and protists also showed remarkable seasonal variations (Figure 3). The results of the Mantel test showed that temperature, DO and salinity had significant effects on bacterial and protist community structure. Among them, the bacterial and nano-protist community structure has the highest correlation with DO (Mantel’s *r*_bac_ = 0.65 and *r*_nano-protist_ = 0.74), while the pico-protist community structure has the highest correlation with temperature (Mantel’s *r* = 0.79). Biological factors also influenced microbial community structure, including PPE and PNF for bacterial communities (Mantel’s *r*_PPE_ = 0.26 and *r*_PNF_ = 0.19), PNF for pico-protist communities (Mantel’s *r* = 0.41), and Syn and PPE for nano-protist communities (Mantel’s *r*_Syn_ = 0.43 and *r*_PPE_ = 0.14). PERMANOVA further revealed significant differences in the communities of all size-fractionated groups in the four seasons (*R*^2^ > 0.17 and *p* < 0.001), but there were no significant differences between depths and habitats (*R*^2^ < 0.02 and *p* > 0.05, Supplementary Table S2). Based on the above results, subsequent analysis focused on the seasonal dynamics of microbial communities.

### 3.3 Seasonal dynamics of microbial community composition

**Figure 4:**
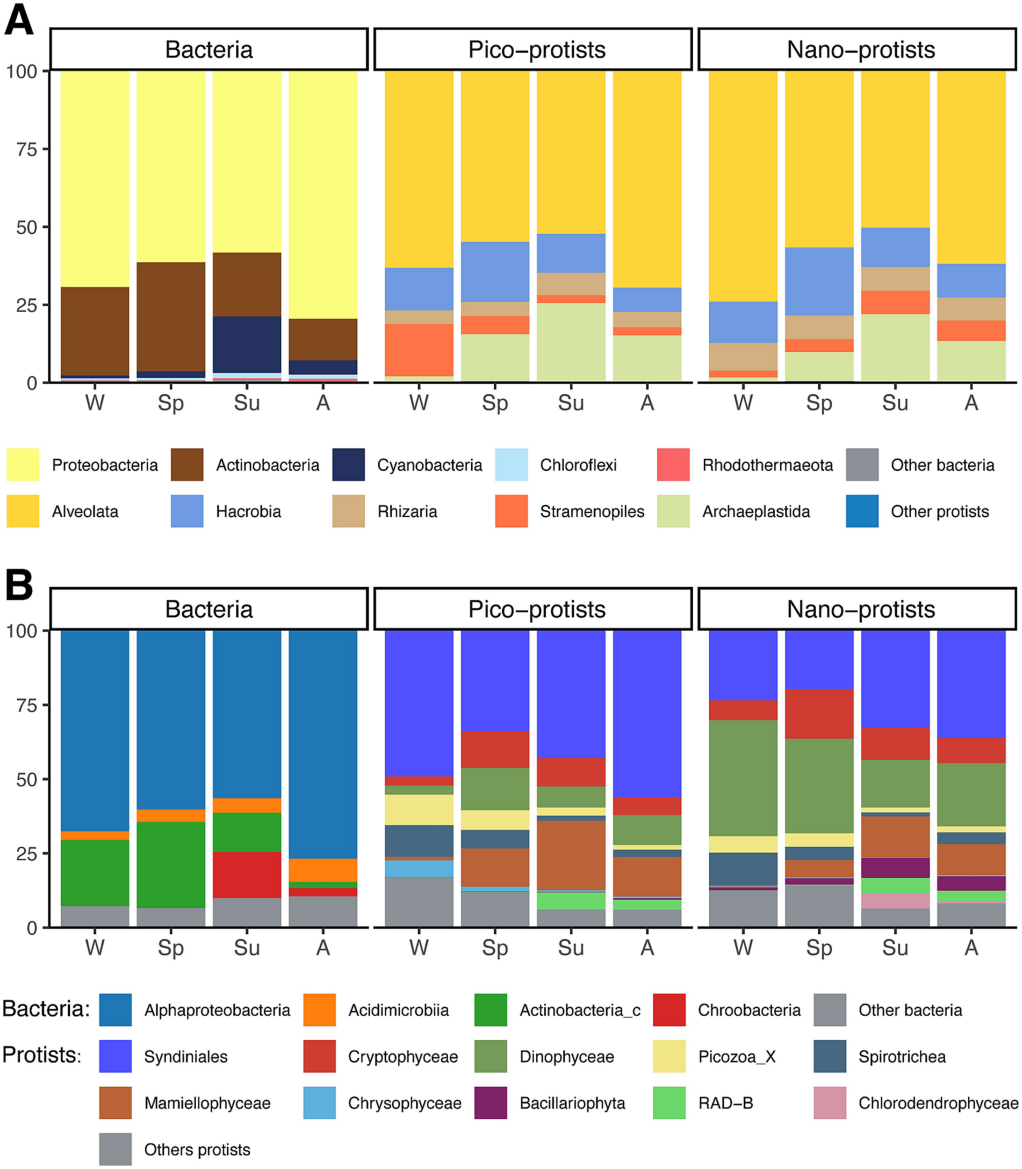
Average relative abundance of major **(A)** bacterial phylum and protist supergroup, and **(B)** class microbial taxonomic groups in four seasons. W, winter; Sp, spring; Su, summer; A, autumn.

The community composition showed pronounced seasonality (Figure 4). The bacterial community was mainly composed of the Alphaproteobacteria class of Proteobacteria phylum (65% ± 10%), followed by Actinobacteria phylum (25% ± 9%). The relative abundance of Alphaproteobacteria peaked in autumn. The Actinobacteria class and the Acidimicrobiia class in the Actinobacteria phylum showed opposite seasonal dynamics, with the relative abundance of the former reaching a minimum in the autumn (2% ± 1%) and the latter reaching a maximum in the autumn (8% ± 2%). Both had opposite responses to seasonal changes in environmental factors such as temperature, salinity, NO_2_-N, NH_4_-N, PPE biomass, and PNF biomass (Supplementary Figure S3). In addition, the average relative abundance of Cyanobacteria was highest in the summer (18% ± 13%) and below 5% in the rest of the season, showing a positive correlation with temperature, salinity, PNF biomass, and HNF biomass, and a negative correlation with nutrient concentrations (Supplementary Figure S3).

Two size-fractionated protist community composition were similar at supergroup level that both were dominated by Alveolata (26–84%), followed by Hacrobia (4–40%) (Figure 4). In the pico-protist community, two supergroups showed complementary seasonal dynamics, where the relative abundance of Stramenopiles peaked in the winter month (17% ± 8%), while Archaeplastida was higher in the other seasons, with the highest proportion in summer month (24% ± 11%). At the class level, the nano-protist communities contained a higher relative abundance of Dinophyceae (28% ± 11%) and a lower abundance of Syndiniales (27% ± 9%) than the pico-protist communities (8% ± 7 and 45% ± 12% respectively), and the other dominant classes differed slightly between the two size-fractionated communities. The response of dominant taxa to environmental factors was also essentially the same for both pico-protists and nano-protists of the same taxon (Supplementary Figures S4, S5). The winter dominant taxa were negatively correlated with temperature, salinity, NO_2_-N, and Syn biomass, and positively correlated with DO, NH_4_-N, and PO_4_-P. The dominant taxa in other seasons were mainly positively correlated with temperature and negatively correlated with PO_4_-P.

**Figure 5:**
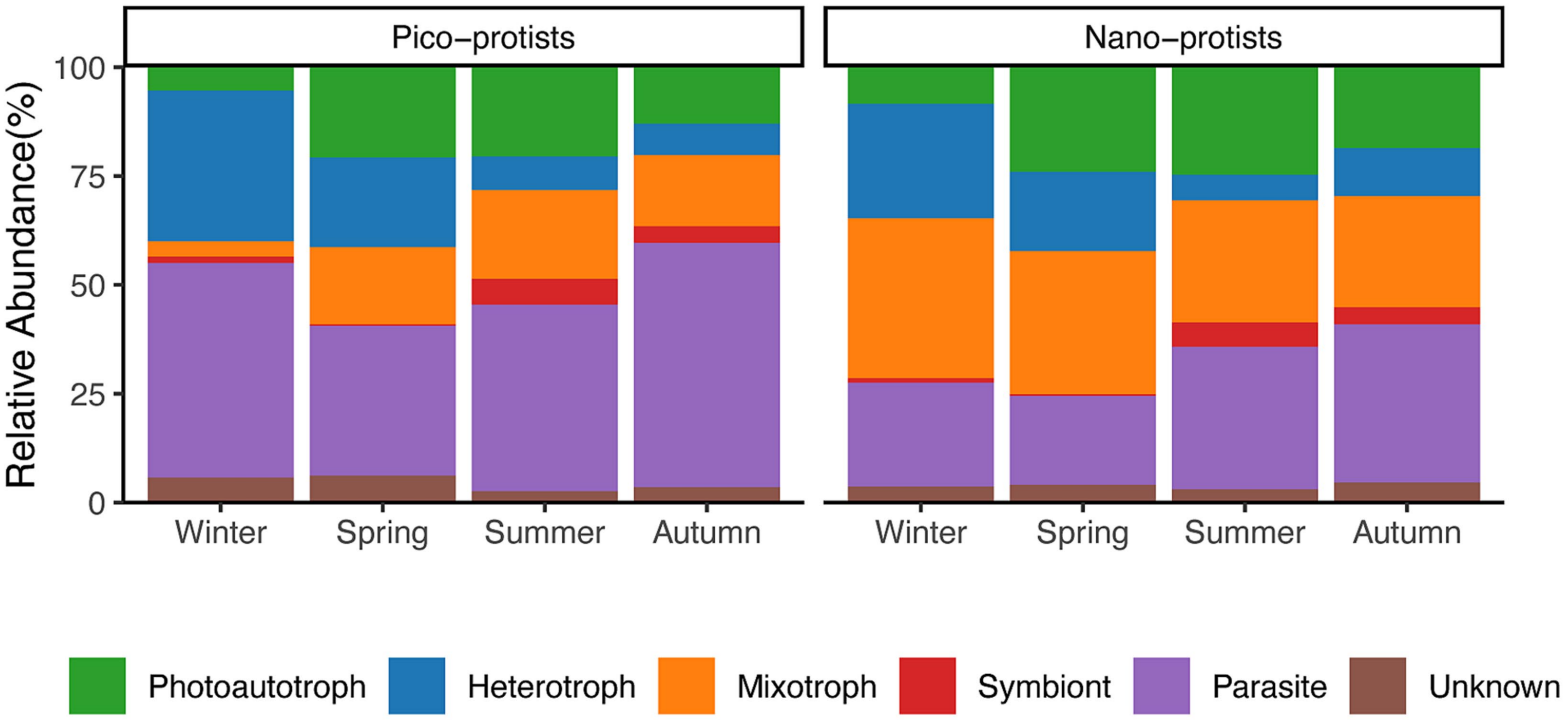
Community compositions of pico-and nano-protists, grouped by trophic modes. Refer to Supplementary Appendix A for trophic mode classification.

The protist communities were trophically divided into photoautotroph, heterotroph, mixotroph, symbiont, parasites, and unknown (Figure 5). From ASV counts, heterotrophs had the highest percentage (31%), parasites and photoautotroph following (24 and 12% respectively), and symbionts were the least abundant (3%). From the relative abundance, parasites were the most abundant in the pico-protist community (46% ± 12%), while mixotrophs were the most abundant in nano-protist communities (31% ± 8%), and symbionts accounted for the lowest percentage in both communities (3% ± 4 and 3% ± 3% respectively). Although the relative abundance of each trophic group differed in the pico-and nano-protist communities, the seasonal trends were generally similar (Supplementary Figures S6, S7). Heterotrophs were most abundant in winter (9–69%), showing negative correlations with temperature, salinity, Chl*a*, and Syn biomass, and positive correlations with PO_4_-P, NO_3_-N, and NH_4_-N. Parasites were abundant in autumn and winter (16–79%), and the correlations with other environmental factors were almost exactly opposite to those of the heterotrophs, except for no significant correlation with PO_4_-P. Photoautotrophs, on the other hand, were relatively abundant in the warmer summer and autumn (6–45%) and were negatively correlated with PO_4_-P. Seasonal dynamics of the mixotrophs differed between the two size-fractionated communities. Pico-mixotrophs were lowest in winter (4% ± 3%) and showed positive correlations with temperature and salinity, and negative correlations with phosphate and DO. Nano-mixotrophs, in contrast, were most abundant in winter (37% ± 7%), and consisted mainly of the red tide species *Heterocapsa rotundata* (25% ± 5%), which showed the opposite environmental correlation to the pico-mixotroph.

### 3.4 Seasonal structure and topological characteristics of microbial networks

**Figure 6:**
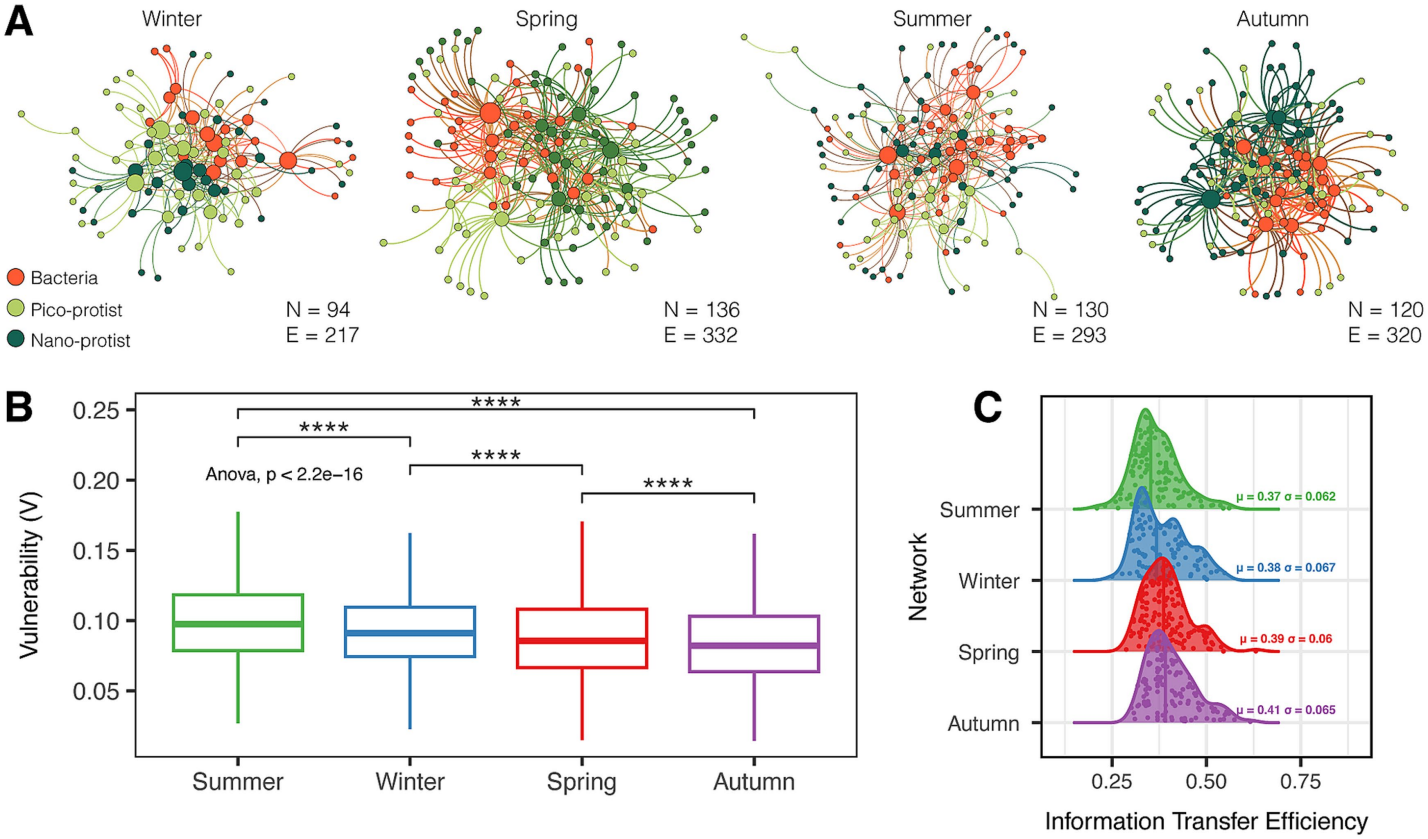
Seasonal interdomain network patterns, network analysis of robustness, and information transfer efficiency. **(A)** The interdomain network was inferred using SpiecEasi analysis with bacteria, pico-, and nano-protist represented by node colors. N, number of ASVs; E, number of edges. **(B)** Vulnerability (V) inferred from randomized attack robustnes, and the significance *p* value of the difference in vulnerability. **(C)** Network seasonal efficiency distribution curves in increasing order of average efficiency, with dots representing each node in the network and labeled with the mean (*μ*) and standard deviation (*σ*) of each curve. Significance level: *p < 0.05; **p < 0.01; ***p < 0.001; ****p < 0.0001.

To evaluate the structural properties of seasonal microbial food webs, bacteria-pico-protist-nano-protist interdomain networks (BPN networks) were constructed using the SpiecEasi method (Figure 6; Table 1). Comparing the networks in the four seasons, the autumn network had the lowest modularity coefficient, average path length, the highest average degree, and clustering coefficient, and was also the most robust (*V*_mean_ = 0.085) and nodal efficient in information transfer (*μ* = 0.41). The summer network was completely opposite, vulnerable (*V*_mean_ = 0.100), and inefficient in the transfer of information (μ = 0.37).

**Table 1:** Topological properties of the bacteria-pico-protists-nano-protists interdomain networks in different seasons.

Season	Winter	Spring	Summer	Autumn
Modularity	0.377	0.421	0.449	0.369
Average path length	2.946	2.806	3.054	2.716
Average degree	2.372	3.018	2.790	3.153
Graph diameter	6	5	7	5
Graph density	0.013	0.014	0.013	0.016
Clustering coefficient	0.126	0.120	0.137	0.167
Degree centralization	0.080	0.192	0.130	0.163
Small world index	19.321	15.115	17.441	18.299

There were no significant differences in the specific contributions of bacteria, pico-protists, and nano-protists to the structural properties of the BPN networks, such as absolute edge weight, degree, and betweenness (Supplementary Figure S8). The network had the highest percentage of nano-protists (34–52%), followed by pico-protists (46–28%), and the lowest by bacteria (20–32%). Network keystone species analysis showed that the nano-protist Dinophyceae class were keystone species in all four seasons, and the pico-protist MAST-4 class in winter, spring, and summer (Supplementary Table S3). These results indicated that protists were important foundational components of the interdomain network structure.

The role of bacteria in the network was non-negligible. In BPN networks, although bacteria constituted a small percentage, they were mostly keystone species (33–55% of the keystone species in the networks). In particular, the Rhodobacteraceae family were keystone species in all four seasons, and the Cyanobacteria in spring, summer, and autumn (Supplementary Table S3). It was also worth noting that the network analysis without distinguishing seasons revealed that the inclusion of bacteria improved the robustness and information transfer efficiency of the networks (Supplementary Figure S10). The bacteria-nano-protist interdomain network (BN network) was the most rubust and efficient among the interdomain networks, followed by the BPN network and the bacteria-pico-protist interdomain network (BP network). Therein, the average edge weights and degrees of bacteria were significantly higher than those of protists. In summary, these results highlighted crucial and highly connected keystone roles of bacteria in enhancing the robustness and efficiency of the Sansa Bay protists networks.

### 3.5 Drivers of microbial network structure

**Figure 7:**
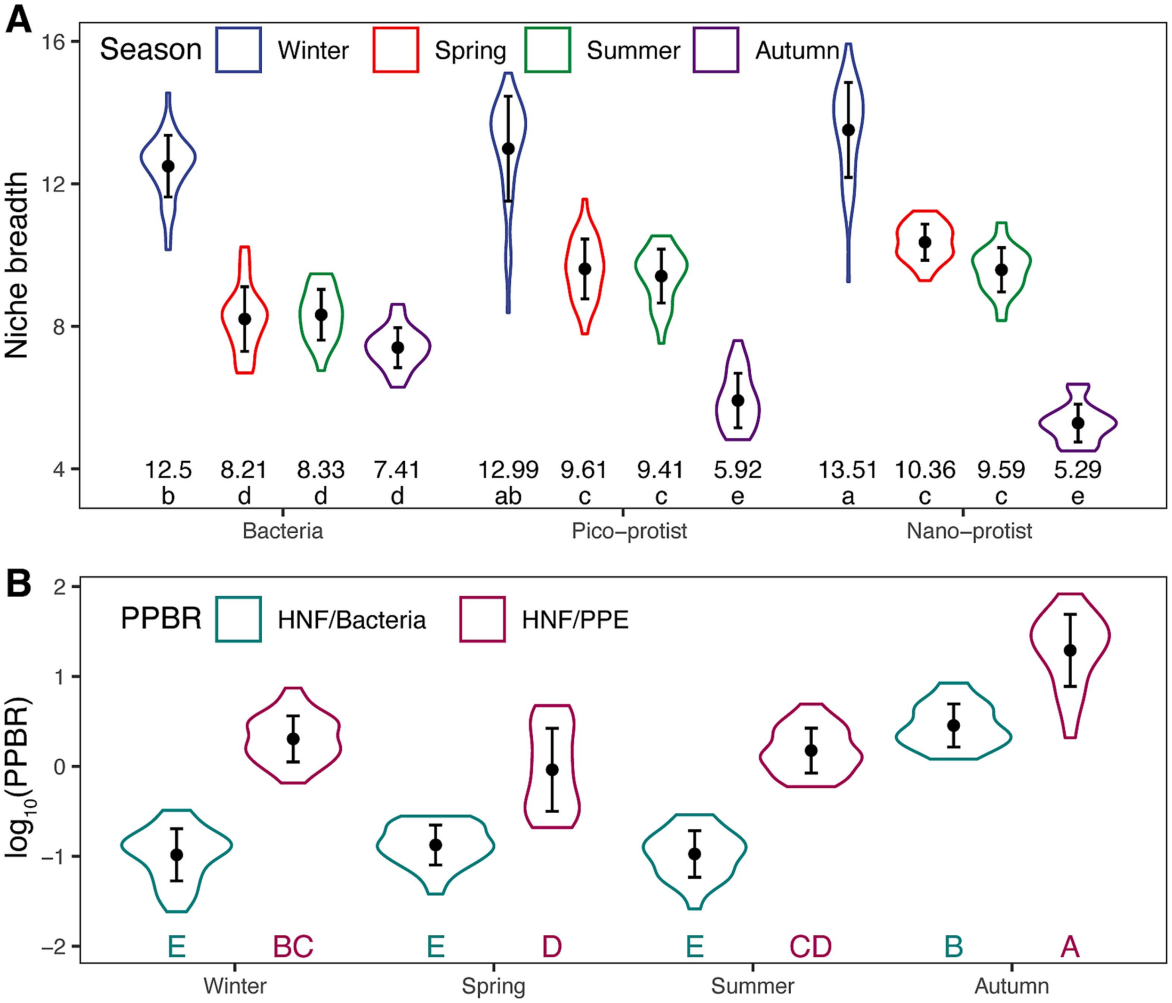
Seasonal **(A)** community level ecological niche breadth and **(B)** predator/prey biomass ratio (PPBR). Different letters indicate statistically significant differences (*p* < 0.05) based on LSD tests; groups sharing the same letter are not significantly different. The numbers below the box indicate the mean values.

To investigate the reasons for the stability and efficiency of the microbial network in autumn, we calculated community-level niche breadth, which can reflect the community’s ability to adapt to the environment, and also used trophic transfer efficiency as represented by the PPBR. The microbial community niche breadth of all three taxa in autumn was the lowest of the year (Figure 7A), indicating that the microbial communities had a narrow environmental tolerance in autumn, which promoted the functional specialization of the community. Furthermore, complex trophic relationships existed between bacteria and nano-protists, and between pico-protists and nano-protists. The results of PPBR showed that the ratio of biomass of HNF to bacteria was significantly lower than that of HNF to PPE, with the ratio of biomass of HNF to PPE in autumn being significantly higher than that in other seasons (Figure 7B). These suggested that trophic transfer efficiency between pico-and nano-sized organisms was particularly high in autumn.

**Figure 8:**
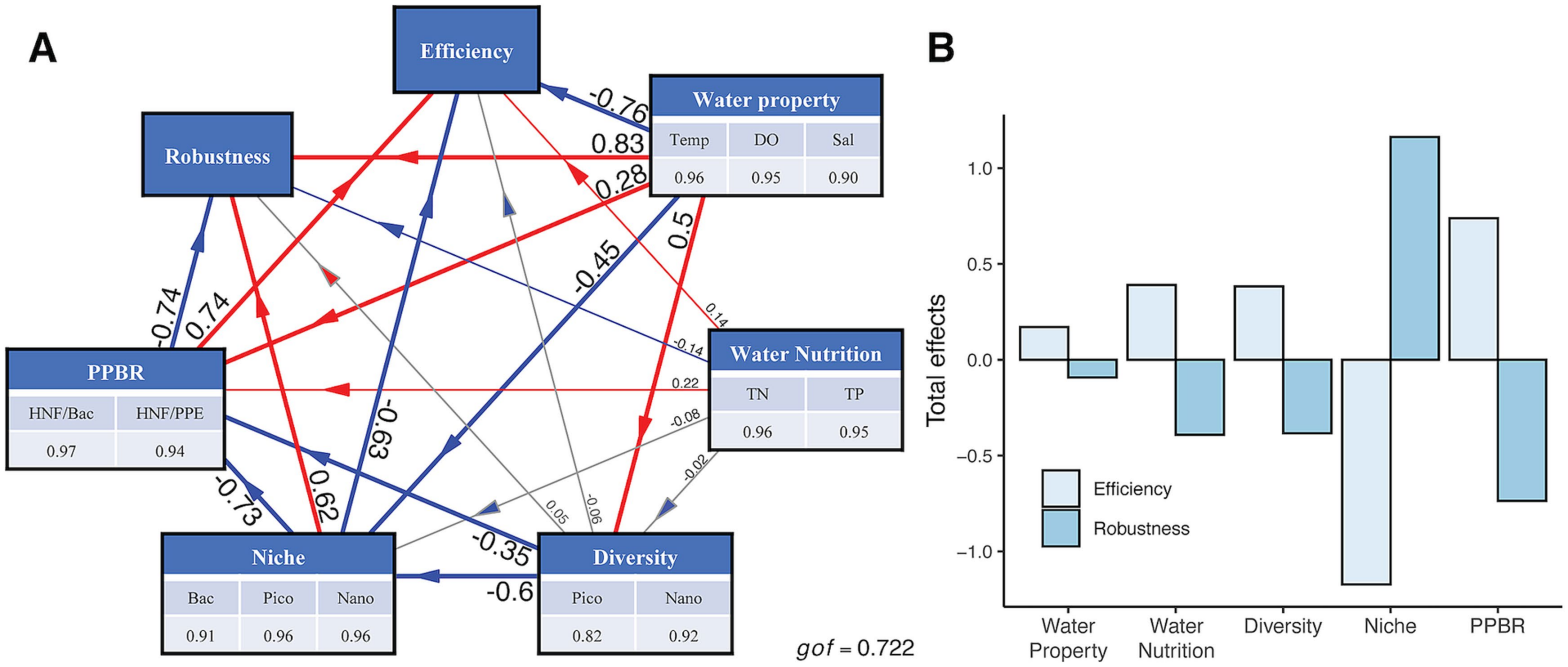
Contribution of biotic and abiotic factors to bacteria-pico-protists-nano-protists interdomain network robustness and efficiency. **(A)** Partial least squares path model (PLS-PM). Each long box represents a latent variable and each parameter in a long box represents an explicit variable and its loading. Red and blue paths represent paths with significant positive and negative impacts, respectively; gray paths are not significant. Numbers represent path coefficients after 1,000 bootstraps. “gof” indicates the goodness of fit. **(B)** The total effects of the latent variables on interdomain network robustness and efficiency. Temp, Temperature; DO, dissolved oxygen; Sal, salinity; TN, total nitrogen; TP, total phosphorus; PPBR, the predator/prey biomass ratio; Bac, bacteria; Pico, pico-protists; Nano, nano-protists.

Based on the previous results, key water property factors (temperature, DO, and salinity), water nutrition (TN and TP), and related biological factors (diversity, community-level niche breadth, PPBR) that shaped the microbial community structure were selected to construct a PLS-PM model. This model was used to investigate the direct and indirect drivers influencing microbial food web stability and the efficiency of information transfer within Sansha Bay (*gof* = 0.722, Figure 8A; Supplementary Table S4). Water property was found to significantly influence not only microbial community diversity (path coefficient = 0.50, *p* < 0.01) and community-level niche breadth (path coefficient = −0.45, *p* < 0.01), but also microbial network robustness (path coefficient = 0.83, *p* < 0.01) and information transfer efficiency (path coefficient = −0.76, *p* < 0.01). The influence of water nutrition on microbial networks was weaker than that of water property and was completely opposite. Considering the total effects, the drivers demonstrated opposing impacts on microbial network robustness and information transfer efficiency (Figure 8B). Among all factors, community-level niche had the strongest influence on microbial network structure, followed by PPBR, and then water nutrition.

## 4 Discussion

### 4.1 Differences in the response of two sized protist communities to seasonal changed

The α-diversity of pico-protist and nano-protist communities showed similar seasonal trends and structure (Figure 2; Supplementary Figure S9), whereas there were differences in composition (Figure 4), in particular the proportion of parasites and mixotrophs in the communities. Similar to the results of the *Tara* Oceans expedition for the oceans (de Vargas et al., 2015a), the pico-protist communities had a higher percentage of parasites compared to the nano-protist communities (Figure 5). On the one hand, this may be inflated due to the higher rDNA copy number in some marine alveolate lineages, specifically the clonal abundance of Syndiniales, the dominant class of the pico-community, overestimates the cellular abundance (Not et al., 2009; Massana, 2011). On the other hand, the parasites released hundreds of small, non-phagocytic dinospores into the water column after killing their host (Guillou et al., 2008; Siano et al., 2011). However, because the parasite hosts encompass a wide range of organisms from flagellates to fish, even studies suggesting that Syndiniales may not always be unequivocally host-specific, it was difficult to find a clear association between the parasites and the potential host and to elucidate the drivers of seasonal dynamics of parasites (Guillou et al., 2008; Käse et al., 2021). In addition, the trophic modes of protists may be flexible and influenced by environmental factors (Flynn et al., 2019). We acknowledged that categorizing taxa into discrete functional groups may oversimplify the ecological complexity and plasticity inherent in protists.

Mixotrophs are important components of microbial food webs (Stoecker et al., 2017). Previous studies confirmed that mixotrophic protists could obtain nutrients through direct ingestion of bacteria or algae (Flynn et al., 2019), and coastal PNFs were important grazers of Synechococcus populations (Tsai et al., 2007; Chan et al., 2009). This trophic relationship likely accounts for the significant correlations observed between nano-protist communities and *Synechococcus* biomass, and between bacterial communities and PNF biomass (Figure 3D). Some mixotrophic protists may also pose risks to aquaculture systems (Flynn et al., 2018). For example, the mixotrophic flagellate *Heterocapsa pygmaea* (Millette et al., 2017), identified as a keystone species in the spring, summer, and autumn interdomain networks (Supplementary Table S3), was the dominant species in a harmful algal bloom near Sansha Bay (Wu et al., 2022). Another mixotroph, *Karlodinium veneficum*, a keystone species in the autumn interdomain network (Supplementary Table S3), is known to cause fish kills during its blooms (Place et al., 2012). However, no algal blooms or fish kills occurred during our sampling, despite the relative abundance of *Heterocapsa* genus exceeded 20% in winter and the relative abundance *Karlodinium veneficum* exceeded 10% at several mariculture stations. The macroalga *Gracilaria lemaneiformis* widely cultured in Sansha Bay, has been shown to effectively inhibit harmful algal bloom formation, likely through trophic competition, allelopathic interactions (Yang et al., 2015)., and shading effects that suppress microalgal growth (Xie et al., 2021).

### 4.2 Habitat specialists promoting robust and efficient microbial networks

It is generally assumed that network efficiency varied inversely with robustness because edges that contain more connections between nodes make the network more resilient to attacks, but less efficient at creating the shortest paths between two nodes (Rodrigue, 2020). However, in our study, the autumn microbial network showed the highest robustness and the highest efficiency (Figure 6). The PLS-PM results revealed that the overwhelming effects of community-level niche breadth on the network’s robustness and efficiency than environmental factors (Figure 8). Given that protists had the narrowest ecological niche breadth in autumn (Figure 7A), we speculated that the remarkable specialization of protists in autumn was an important driver of rubust and highly efficient interdomain microbial networks.

Multiple environmental ecology studies have demonstrated that microbial specialists play a crucial role in biological interactions (Finke and Snyder, 2008; Yan et al., 2022; Li et al., 2024; Zhou et al., 2024). Synthetic community experiments further revealed the mechanism of microbial specialists’ roles. Specialists established a clearer functional division of labor by utilizing specific resources, such as specific carbon sources, nitrogen sources, or microhabitats (Huang et al., 2019). Specialization reduced the competitive pressure caused by overlapping resources, which made the species interaction more reciprocal or neutral, thus enhancing the stability of the network structure (Bai et al., 2015). When environmental stress increased, specialists played a more crucial role in stabilizing ecological networks (Li et al., 2023). Moreover, specialization also reduced redundant connections and improved the efficiency of information transfer (Tsoi et al., 2019; Wang et al., 2024).

Differently from protists, bacteria contributed to BPN networks not only by specialization. On the one hand, season-independent network construction revealed that the inclusion of bacteria contributed to a robust microbial network, evidenced that bacteria worked to connect members of the community and thereby exchange metabolites or were consumed. For example, Rhodobacteraceae, identified as key species in all four seasonal BPN networks (Supplementary Table S3), were known to have multifaceted and mutually infochemical exchanges with phytoplankton, such as diatoms and dinoflagellate, and their interactions dynamically change according to the physiological state of the phytoplankton (Amin et al., 2015; Seymour et al., 2017). On the other hand, the community diversity of more generalist bacteria was excluded from the PLS-PM analysis due to low loadings. This implied that a select few bacteria, which possess specific relationships with phytoplankton and protist communities, emerged as the keystone species of the interdomain microbial network. However, most generalist bacteria contributed minimally to the interdomain network structure, as their increased diversity did not translate into more efficient resource utilization compared to that of specialists (Finke and Snyder, 2008).

### 4.3 Synergistic effects of integrated multi-trophic mariculture and the natural environment

Despite the presence of macroalgae farms that absorbed a portion of the excess nutrients from cage cultures, the waters of Sansha Bay remained persistently eutrophic (Xie et al., 2020). PLS-PM results revealed that the total effect of water nutrients (TN and TP) on microbial network structure exceeded water quality property (Figure 8). This anthropogenic eutrophication resulted in significant changes in microbial community structure (Kiersztyn et al., 2019; Chen et al., 2024). PLS-PM results also showed significant effects of HNF predation on network robustness and information transfer efficiency (Figure 8), suggesting that predatory relationships were central to microbial food webs. HNFs as major predators of pico-sized microorganisms were controlled by food supply (Lin et al., 2016), playing a top-down control role in the microbial food web. Such biological interactions were critical for maintaining the stability of bacterial communities (Liu et al., 2022).

The community structure of marine microorganisms was determined by various abiotic and biotic environmental factors such as temperature, salinity, and nutrients (Logares et al., 2009; Sunagawa et al., 2015; Dai et al., 2022). In cultured waters, dissolved organic matter (DOM) released from macroalgae and fish culture was also a key factor influencing the microbial community structure (Yang et al., 2015; Luo et al., 2024; Ou et al., 2024). In Sansha Bay, year-round cultured *Gracilaria lemaneiformis* provided a steady input of polysaccharides while inhibiting phytoplankton blooms (Yang et al., 2015; Ou et al., 2024). After *Saccharina japonica* was harvested in May, there was less complex DOM remaining in autumn, and the resources available to microbes tended to be simpler, reducing the microbial community ecological niche (Tanentzap et al., 2019; Huang et al., 2024). *Larimichthys crocea* also experienced a rapid growth period in autumn (midway through the 8–13 month rearing cycle), and it had a continuous and steady input of metabolites, creating a predictable pattern of nutrient supply (Luo et al., 2024). Moreover, compared to summer, there were barely any typhoons in autumn, the hydrological conditions were stable, and physical perturbations had little effect on the microbial community structure (Nguyen et al., 2021).

## 5 Conclusion

This study elucidated the impact of mariculture activities and seasonal variations on marine microbial diversity and interdomain microbial networks in Sansha Bay. Our results show that pico-protists were predominantly composed of parasites, whereas nano-protists mainly mixotrophs. Despite the presence of multiple red tide species and parasitic taxa, the integrated multi-trophic mariculture system in the study area may help reduce the risk of algal blooms or fish kills events. The microbial community showed pronounced seasonality, primarily driven by temperature, dissolved oxygen, and salinity, alongside marked shifts in interdomain network structures. The autumn network was both the most robust and efficient, associated with narrower niche breadths among protists, suggesting a strong role of specialization in stabilizing microbial interactions. These findings highlight the importance of ecological niche differentiation and microbial interactions in maintaining network structure and function under environmental and anthropogenic influences. Our work contributes to the understanding of microbial food web dynamics in coastal aquaculture systems and provided a theoretical basis for enhancing the important role of microbial food webs in the biogeochemical cycle and energy flow through the optimization of maricultural modes.

## Data Availability

The original contributions presented in the study are publicly available. This data can be found here: https://www.ncbi.nlm.nih.gov, accession number PRJNA1252866.

## Footnotes

^1^ http://mothur.org/wiki/miseq_sop/

## References

[ref1] Adl S. M. Bass D. Lane C. E. Lukes J. Schoch C. L. Smirnov A.. (2019). Revisions to the classification, nomenclature, and diversity of eukaryotes. J. Eukaryot. Microbiol. 66, 4–119. doi: 10.1111/jeu.12691, PMID: 30257078 PMC6492006

[ref2] Adl S. M. Simpson A. G. B. Lane C. E. Lukeš J. Bass D. Bowser S. S.. (2012). The revised classification of eukaryotes. J. Eukaryot. Microbiol. 59, 429–514. doi: 10.1111/j.1550-7408.2012.00644.x, PMID: 23020233 PMC3483872

[ref3] Amin S. A. Hmelo L. R. van Tol H. M. Durham B. P. Carlson L. T. Heal K. R.. (2015). Interaction and signalling between a cosmopolitan phytoplankton and associated bacteria. Nature 522, 98–101. doi: 10.1038/nature14488, PMID: 26017307

[ref4] Azam F. Malfatti F. (2007). Microbial structuring of marine ecosystems. Nat. Rev. Microbiol. 5, 782–791. doi: 10.1038/nrmicro1747, PMID: 17853906

[ref5] Bachy C. Hehenberger E. Ling Y.-C. Needham D. M. Strauss J. Wilken S.. (2022). “Marine Protists: a hitchhiker’s guide to their role in the marine microbiome” in The marine microbiome. eds. Stal L. J. Cretoiu M. S. (Cham: Springer International Publishing), 159–241.

[ref6] Bai Y. Müller D. B. Srinivas G. Garrido-Oter R. Potthoff E. Rott M.. (2015). Functional overlap of the Arabidopsis leaf and root microbiota. Nature 528, 364–369. doi: 10.1038/nature16192, PMID: 26633631

[ref7] Baldauf S. L. (2003). The deep roots of eukaryotes. Science 300, 1703–1706. doi: 10.1126/science.1085544, PMID: 12805537

[ref8] Berry D. Widder S. (2014). Deciphering microbial interactions and detecting keystone species with co-occurrence networks. Front. Microbiol. 5:219. doi: 10.3389/fmicb.2014.00219, PMID: 24904535 PMC4033041

[ref9] Caron D. A. Alexander H. Allen A. E. Archibald J. M. Armbrust E. V. Bachy C.. (2017). Probing the evolution, ecology and physiology of marine protists using transcriptomics. Nat. Rev. Microbiol. 15, 6–20. doi: 10.1038/nrmicro.2016.160, PMID: 27867198

[ref10] Cavicchioli R. Ripple W. J. Timmis K. N. Azam F. Bakken L. R. Baylis M.. (2019). Scientists’ warning to humanity: microorganisms and climate change. Nat. Rev. Microbiol. 17, 569–586. doi: 10.1038/s41579-019-0222-5, PMID: 31213707 PMC7136171

[ref11] Chan Y.-F. Tsai A.-Y. Chiang K.-P. Hsieh C. (2009). Pigmented nanoflagellates grazing on Synechococcus: seasonal variations and effect of flagellate size in the coastal ecosystem of subtropical western Pacific. Microb. Ecol. 58, 548–557. doi: 10.1007/s00248-009-9569-x, PMID: 19655080

[ref12] Charpy L. Blanchot J. (1998). Photosynthetic picoplankton in French Polynesian atoll lagoons: estimation of taxa contribution to biomass and production by flow cytometry. Mar. Ecol. Prog. Ser. 162, 57–70. doi: 10.3354/meps162057

[ref13] Chen C. Li P. Yin M. Wang J. Sun Y. Ju W.. (2023). Deciphering characterization of seasonal variations in microbial communities of marine ranching: diversity, co-occurrence network patterns, and assembly processes. Mar. Pollut. Bull. 197:115739. doi: 10.1016/j.marpolbul.2023.115739, PMID: 37925991

[ref14] Chen Q. Liu Y. Zhang M. Lin K. Wang Z. Liu L. (2024). Seasonal responses of microbial communities to water quality variations and interaction of eutrophication risk in Gehu Lake. Sci. Total Environ. 955:177199. doi: 10.1016/j.scitotenv.2024.177199, PMID: 39471940

[ref15] Chen C.-C. Shiah F.-K. Chiang K.-P. Gong G.-C. Kemp W. M. (2009). Effects of the Changjiang (Yangtze) river discharge on planktonic community respiration in the East China Sea. J. Geophys. Res. Oceans 114:C03005. doi: 10.1029/2008JC004891, PMID: 39712630

[ref16] Dai T. Wen D. Bates C. T. Wu L. Guo X. Liu S.. (2022). Nutrient supply controls the linkage between species abundance and ecological interactions in marine bacterial communities. Nat. Commun. 13:175. doi: 10.1038/s41467-021-27857-6, PMID: 35013303 PMC8748817

[ref17] de Vargas C. Audic S. Henry N. Decelle J. Mahé F. Logares R.. (2015a). Eukaryotic plankton diversity in the sunlit ocean. Science 348:1261605. doi: 10.1126/science.1261605, PMID: 25999516

[ref18] de Vargas C. Tara Oceans Consortium C. Tara Oceans Expedition P. (2015b). Total V9 rDNA information organized at the OTU level (database W5). PANGAEA.

[ref19] Deutschmann I. M. Krabberød A. K. Latorre F. Delage E. Marrasé C. Balagué V.. (2023). Disentangling temporal associations in marine microbial networks. Microbiome 11:83. doi: 10.1186/s40168-023-01523-z, PMID: 37081491 PMC10120119

[ref9001] Edgar R. C. Haas B. J. Clemente J. C. Quince C. Knight R. (2011). UCHIME improves sensitivity and speed of chimera detection. Bioinform. 27, 2194–2200. doi: 10.1093/bioinformatics/btr381 PMC3150044 21700674

[ref20] Edwards M. Richardson A. J. (2004). Impact of climate change on marine pelagic phenology and trophic mismatch. Nature 430, 881–884. doi: 10.1038/nature02808, PMID: 15318219

[ref21] Finke D. L. Snyder W. E. (2008). Niche partitioning increases resource exploitation by diverse communities. Science 321, 1488–1490. doi: 10.1126/science.1160854, PMID: 18787167

[ref22] Flemming H.-C. Wuertz S. (2019). Bacteria and archaea on earth and their abundance in biofilms. Nat. Rev. Microbiol. 17, 247–260. doi: 10.1038/s41579-019-0158-9, PMID: 30760902

[ref23] Flynn K. J. Mitra A. Anestis K. Anschütz A. A. Calbet A. Ferreira G. D.. (2019). Mixotrophic protists and a new paradigm for marine ecology: where does plankton research go now? J. Plankton Res. 41, 375–391. doi: 10.1093/plankt/fbz026

[ref24] Flynn K. J. Mitra A. Glibert P. M. Burkholder J. M. (2018). “Mixotrophy in harmful algal blooms: by whom, on whom, when, why, and what next,” in Global ecology and oceanography of harmful algal blooms, eds. Glibert P. M. Berdalet E. Burford M. A. Pitcher G. C. Zhou M. (Cham: Springer International Publishing), 113–132. (Accessed April 28, 2025).

[ref25] Friedman J. Alm E. J. (2012). Inferring correlation networks from genomic survey data. PLoS Comput. Biol. 8:e1002687. doi: 10.1371/journal.pcbi.1002687, PMID: 23028285 PMC3447976

[ref26] Fuhrman J. A. (2009). Microbial community structure and its functional implications. Nature 459, 193–199. doi: 10.1038/nature08058, PMID: 19444205

[ref27] Fuhrman J. A. Cram J. A. Needham D. M. (2015). Marine microbial community dynamics and their ecological interpretation. Nat. Rev. Microbiol. 13, 133–146. doi: 10.1038/nrmicro3417, PMID: 25659323

[ref28] García-Comas C. Sastri A. R. Ye L. Chang C.-Y. Lin F.-S. Su M.-S.. (2016). Prey size diversity hinders biomass trophic transfer and predator size diversity promotes it in planktonic communities. Proc. R. Soc. B Biol. Sci. 283:20152129. doi: 10.1098/rspb.2015.2129, PMID: 26865298 PMC4760158

[ref29] Guillou L. Viprey M. Chambouvet A. Welsh R. M. Kirkham A. R. Massana R.. (2008). Widespread occurrence and genetic diversity of marine parasitoids belonging to Syndiniales (Alveolata). Environ. Microbiol. 10, 3349–3365. doi: 10.1111/j.1462-2920.2008.01731.x, PMID: 18771501

[ref9002] Guillou L. Bachar D. Audic S. Bass D. Berney C. Bittner L.. (2012). The Protist Ribosomal Reference database (PR2): a catalog of unicellular eukaryote Small Sub-Unit rRNA sequences with curated taxonomy. Nucleic Acids Res. 41, D597–D604. doi: 10.1093/nar/gks1160 23193267 PMC3531120

[ref30] Guo X. Liu Q. Lin X. Zheng X. Huang C. Pang M.. (2023). Water mass-driven multiple ecological effects determine the biodiversity and community assembly of microbial flagellates in subtropic-tropic marginal seas of China. Estuar. Coast. Shelf Sci. 280:108166. doi: 10.1016/j.ecss.2022.108166

[ref31] Guo X. Wu L. Huang L. (2020). Spatiotemporal patterns in diversity and assembly process of marine Protist communities of the Changjiang (Yangtze River) plume and its adjacent waters. Front. Microbiol. 11:579290. doi: 10.3389/fmicb.2020.579290, PMID: 33123109 PMC7573215

[ref32] Huang A. C. Jiang T. Liu Y.-X. Bai Y.-C. Reed J. Qu B.. (2019). A specialized metabolic network selectively modulates Arabidopsis root microbiota. Science 364:eaau6389. doi: 10.1126/science.aau6389, PMID: 31073042

[ref33] Huang H. Zan S. Shao K. Chen H. Fan J. (2024). Spatial distribution characteristics and interaction effects of DOM and microbial communities in kelp cultivation areas. Sci. Total Environ. 920:170511. doi: 10.1016/j.scitotenv.2024.170511, PMID: 38309352

[ref34] Iyer S. Killingback T. Sundaram B. Wang Z. (2013). Attack robustness and centrality of complex networks. PLoS One 8:e59613. doi: 10.1371/journal.pone.0059613, PMID: 23565156 PMC3615130

[ref35] Jürgens K. Massana R. (2008). “Protistan grazing on marine Bacterioplankton” in Microbial ecology of the Oceans, 383–441.

[ref36] Käse L. Metfies K. Neuhaus S. Boersma M. Wiltshire K. H. Kraberg A. C. (2021). Host-parasitoid associations in marine planktonic time series: can metabarcoding help reveal them? PLoS One 16:e0244817. doi: 10.1371/journal.pone.0244817, PMID: 33411833 PMC7790432

[ref37] Kiersztyn B. Chróst R. Kaliński T. Siuda W. Bukowska A. Kowalczyk G.. (2019). Structural and functional microbial diversity along a eutrophication gradient of interconnected lakes undergoing anthropopressure. Sci. Rep. 9:11144. doi: 10.1038/s41598-019-47577-8, PMID: 31366993 PMC6668414

[ref38] Kim J. Cui Y. Nam K.-H. Lee J.-W. Kim J.-G. Chun S.-J. (2025). Microbial generalists as keystone species: constructing core network modules in the anthosphere of twelve diverse wild plant species. Environ. Microb. 20:6. doi: 10.1186/s40793-025-00666-w, PMID: 39810271 PMC11730483

[ref39] Kirkham A. R. Lepère C. Jardillier L. E. Not F. Bouman H. Mead A.. (2013). A global perspective on marine photosynthetic picoeukaryote community structure. ISME J. 7, 922–936. doi: 10.1038/ismej.2012.166, PMID: 23364354 PMC3635238

[ref40] Kneitel J. M. Chase J. M. (2004). Trade-offs in community ecology: linking spatial scales and species coexistence. Ecol. Lett. 7, 69–80. doi: 10.1046/j.1461-0248.2003.00551.x

[ref41] Kozich J. J. Westcott S. L. Baxter N. T. Highlander S. K. Schloss P. D. (2013). Development of a dual-index sequencing strategy and curation pipeline for analyzing amplicon sequence data on the MiSeq Illumina sequencing platform. Appl. Environ. Microbiol. 79, 5112–5120. doi: 10.1128/AEM.01043-13, PMID: 23793624 PMC3753973

[ref42] Kurtz Z. D. Müller C. L. Miraldi E. R. Littman D. R. Blaser M. J. Bonneau R. A. (2015). Sparse and compositionally robust inference of microbial ecological networks. PLoS Comput. Biol. 11:e1004226. doi: 10.1371/journal.pcbi.1004226, PMID: 25950956 PMC4423992

[ref43] Landry M. R. Schroer W. F. (2019). “Microbial Loops” in Encyclopedia of ocean sciences. eds. Cochran J. K. Bokuniewicz H. J. Yager P. L.. Third ed (Oxford: Academic Press), 739–745.

[ref44] Latora V. Marchiori M. (2001). Efficient behavior of small-world networks. Phys. Rev. Lett. 87:198701. doi: 10.1103/PhysRevLett.87.198701, PMID: 11690461

[ref45] Lee S. Fuhrman J. A. (1987). Relationships between biovolume and biomass of naturally derived marine Bacterioplankton. Appl. Environ. Microbiol. 53, 1298–1303. doi: 10.1128/aem.53.6.1298-1303.1987, PMID: 16347362 PMC203858

[ref46] Li C. Jin L. Zhang C. Li S. Zhou T. Hua Z.. (2023). Destabilized microbial networks with distinct performances of abundant and rare biospheres in maintaining networks under increasing salinity stress. iMeta 2:e79. doi: 10.1002/imt2.79, PMID: 38868331 PMC10989821

[ref47] Li S. Yan X. Abdullah Al M. Ren K. Rensing C. Hu A.. (2024). Ecological and evolutionary processes involved in shaping microbial habitat generalists and specialists in urban park ecosystems. mSystems 9:e0046924. doi: 10.1128/msystems.00469-24, PMID: 38767347 PMC11237591

[ref48] Lin S. Huang L. Zhu Z. Xiong Y. Lu J. (2016). Distribution of nanoflagellates in five water masses of the East China Sea in autumn and winter. Deep Sea Res. Part II Top. Stud. Oceanogr. 124, 93–99. doi: 10.1016/j.dsr2.2015.02.017

[ref49] Liu C. Cui Y. Li X. Yao M. (2020). Microeco: an R package for data mining in microbial community ecology. FEMS Microbiol. Ecol. 97:fiaa255. doi: 10.1093/femsec/fiaa255, PMID: 33332530

[ref50] Liu M. Liu L. Chen H. Yu Z. Yang J. R. Xue Y.. (2019). Community dynamics of free-living and particle-attached bacteria following a reservoir Microcystis bloom. Sci. Total Environ. 660, 501–511. doi: 10.1016/j.scitotenv.2018.12.414, PMID: 30640117

[ref51] Liu S. Yu H. Yu Y. Huang J. Zhou Z. Zeng J.. (2022). Ecological stability of microbial communities in Lake Donghu regulated by keystone taxa. Ecol. Indic. 136:108695. doi: 10.1016/j.ecolind.2022.108695

[ref52] Logares R. Bråte J. Bertilsson S. Clasen J. L. Shalchian-Tabrizi K. Rengefors K. (2009). Infrequent marine–freshwater transitions in the microbial world. Trends Microbiol. 17, 414–422. doi: 10.1016/j.tim.2009.05.010, PMID: 19726194

[ref53] Luo J. Wang N. Zhu Y. Wu Z. Ye Z. Christakos G.. (2024). Seasonal effects of fish, seaweed and abalone cultures on dissolved organic matter and carbon sequestration potential in Sansha Bay, China. Sci. Total Environ. 945:174144. doi: 10.1016/j.scitotenv.2024.174144, PMID: 38901588

[ref54] Ma Y. Pan Y. Liu Q. Huang L. Zhang W. (2021). Co-occurrence patterns and assembly processes of microeukaryotic communities in a semi-enclosed aquaculture bay. Cont. Shelf Res. 228:104550. doi: 10.1016/j.csr.2021.104550

[ref55] Marie D. Partensky F. Jacquet S. Vaulot D. (1997). Enumeration and cell cycle analysis of natural populations of marine picoplankton by flow cytometry using the nucleic acid stain SYBR green I. Appl. Environ. Microbiol. 63, 186–193. doi: 10.1128/aem.63.1.186-193.1997, PMID: 16535483 PMC1389098

[ref56] Marie D. Simon N. Guillou L. Partensky F. Vaulot D. (2000). Flow cytometry analysis of marine picoplankton, in In living color: Protocols in flow cytometry and cell sorting, eds. Diamond R. A. Demaggio S. (Berlin, Heidelberg: Springer), 421–454 (Accessed October 17, 2023).

[ref57] Massana R. (2011). Eukaryotic picoplankton in surface Oceans. Ann. Rev. Microbiol. 65, 91–110. doi: 10.1146/annurev-micro-090110-102903, PMID: 21639789

[ref58] Mendiburu F. (2010). Agricolae: statistical procedures for agricultural research. R package version 1, 1–8.

[ref59] Millette N. C. Pierson J. J. Aceves A. Stoecker D. K. (2017). Mixotrophy in Heterocapsa rotundata: a mechanism for dominating the winter phytoplankton. Limnol. Oceanogr. 62, 836–845. doi: 10.1002/lno.10470

[ref60] Mo Y. Zhang W. Wilkinson D. M. Yu Z. Xiao P. Yang J. (2021). Biogeography and co-occurrence patterns of bacterial generalists and specialists in three subtropical marine bays. Limnol. Oceanogr. 66, 793–806. doi: 10.1002/lno.11643

[ref61] Moran M. A. (2015). The global ocean microbiome. Science 350:aac8455. doi: 10.1126/science.aac8455, PMID: 26659059

[ref62] Muller E. E. L. (2019). Determining microbial niche breadth in the environment for better ecosystem fate predictions. mSystems 4:e00080-19. doi: 10.1128/mSystems.00080-19, PMID: 31186307 PMC6584867

[ref63] Nguyen J. Lara-Gutiérrez J. Stocker R. (2021). Environmental fluctuations and their effects on microbial communities, populations and individuals. FEMS Microbiol. Rev. 45:fuaa068. doi: 10.1093/femsre/fuaa068, PMID: 33338228 PMC8371271

[ref64] Not F. del Campo J. Balagué V. de Vargas C. Massana R. (2009). New insights into the diversity of marine picoeukaryotes. PLoS One 4:e7143. doi: 10.1371/journal.pone.0007143, PMID: 19787059 PMC2747013

[ref65] Oksanen J. Simpson G. Blanchet F. G. Kindt R. Legendre P. Minchin P. (2022). Vegan community ecology package version 2.6-2 April 2022.

[ref66] Ou X.-L. Ou L.-J. Yang Y.-F. (2024). Bioavailability of dissolved organic matter (DOM) derived from seaweed *Gracilaria lemaneiformis* meditated by microorganisms. Mar. Pollut. Bull. 209:117243. doi: 10.1016/j.marpolbul.2024.117243, PMID: 39522397

[ref67] Pace N. R. (1997). A molecular view of microbial diversity and the biosphere. Science 276, 734–740. doi: 10.1126/science.276.5313.734, PMID: 9115194

[ref68] Pandit S. N. Kolasa J. Cottenie K. (2009). Contrasts between habitat generalists and specialists: an empirical extension to the basic metacommunity framework. Ecology 90, 2253–2262. doi: 10.1890/08-0851.1, PMID: 19739387

[ref69] Place A. R. Bowers H. A. Bachvaroff T. R. Adolf J. E. Deeds J. R. Sheng J. (2012). Karlodinium veneficum—the little dinoflagellate with a big bite. Harmful Algae 14, 179–195. doi: 10.1016/j.hal.2011.10.021

[ref9003] Quast C. Pruesse E. Yilmaz P. Gerken J. Schweer T. Yarza P.. (2012). The SILVA ribosomal RNA gene database project: improved data processing and web-based tools. Nucleic Acids Res. 41, D590–D596. doi: 10.1093/nar/gks1219 23193283 PMC3531112

[ref70] R Core Team (2019). R: A language and environment for statistical computing. Vienna, Austria: R Foundation for Statistical Computing. Available at: https://www.R-project.org/

[ref71] Rodrigue J.-P. (2020). The geography of transport systems. 5th Edn. London: Routledge.

[ref72] Röttjers L. Faust K. (2018). From hairballs to hypotheses–biological insights from microbial networks. FEMS Microbiol. Rev. 42, 761–780. doi: 10.1093/femsre/fuy030, PMID: 30085090 PMC6199531

[ref73] Roume H. Heintz-Buschart A. Muller E. E. L. May P. Satagopam V. P. Laczny C. C.. (2015). Comparative integrated omics: identification of key functionalities in microbial community-wide metabolic networks. NPJ Biofilms Microbiomes 1:15007. doi: 10.1038/npjbiofilms.2015.7, PMID: 28721231 PMC5515219

[ref74] Sanchez G. Trinchera L. Russolillo G. (2024). plspm: Partial least squares path modeling (PLS-PM).

[ref75] Seymour J. R. Amin S. A. Raina J.-B. Stocker R. (2017). Zooming in on the phycosphere: the ecological interface for phytoplankton–bacteria relationships. Nat. Microbiol. 2:17065. doi: 10.1038/nmicrobiol.2017.65, PMID: 28555622

[ref76] Shekarriz E. Chen J. Xu Z. Liu H. (2023). Disentangling the functional role of fungi in cold seep sediment. Microbiol. Spectr. 11:e01978-22. doi: 10.1128/spectrum.01978-22, PMID: 36912690 PMC10100914

[ref77] Siano R. Alves-de-Souza C. Foulon E. Bendif E. M. Simon N. Guillou L.. (2011). Distribution and host diversity of Amoebophryidae parasites across oligotrophic waters of the Mediterranean Sea. Biogeosciences 8, 267–278. doi: 10.5194/bg-8-267-2011

[ref78] Sieburth J. M. Smetacek V. Lenz J. (1978). Pelagic ecosystem structure: heterotrophic compartments of the plankton and their relationship to plankton size fractions. Limnol. Oceanogr. 23, 1256–1263. doi: 10.4319/lo.1978.23.6.1256

[ref79] Sommeria-Klein G. Watteaux R. Ibarbalz F. M. Pierella Karlusich J. J. Iudicone D. Bowler C.. (2021). Global drivers of eukaryotic plankton biogeography in the sunlit ocean. Science 374, 594–599. doi: 10.1126/science.abb3717, PMID: 34709919

[ref80] Stoeck T. Bass D. Nebel M. Christen R. Jones M. D. M. Breiner H.-W.. (2010). Multiple marker parallel tag environmental DNA sequencing reveals a highly complex eukaryotic community in marine anoxic water. Mol. Ecol. 19, 21–31. doi: 10.1111/j.1365-294X.2009.04480.x, PMID: 20331767

[ref81] Stoecker D. K. Hansen P. J. Caron D. A. Mitra A. (2017). Mixotrophy in the marine plankton. Annu. Rev. Mar. Sci. 9, 311–335. doi: 10.1146/annurev-marine-010816-060617, PMID: 27483121

[ref82] Sunagawa S. Coelho L. P. Chaffron S. Kultima J. R. Labadie K. Salazar G.. (2015). Structure and function of the global ocean microbiome. Science 348:1261359. doi: 10.1126/science.1261359, PMID: 25999513

[ref83] Tanentzap A. J. Fitch A. Orland C. Emilson E. J. S. Yakimovich K. M. Osterholz H.. (2019). Chemical and microbial diversity covary in fresh water to influence ecosystem functioning. Proc. Natl. Acad. Sci. 116, 24689–24695. doi: 10.1073/pnas.1904896116, PMID: 31740592 PMC6900631

[ref84] Tsai A.-Y. Chiang K.-P. Chan Y.-F. Lin Y.-C. Chang J. (2007). Pigmented nanoflagellates in the coastal western subtropical Pacific are important grazers on Synechococcus populations. J. Plankton Res. 29, 71–77. doi: 10.1093/plankt/fbl058

[ref85] Tsoi R. Dai Z. You L. (2019). Emerging strategies for engineering microbial communities. Biotechnol. Adv. 37:107372. doi: 10.1016/j.biotechadv.2019.03.011, PMID: 30880142 PMC6710121

[ref86] Wang M. Chen X. Fang Y. Zheng X. Huang T. Nie Y.. (2024). The trade-off between individual metabolic specialization and versatility determines the metabolic efficiency of microbial communities. Cells 15:e5, 63–74. doi: 10.1016/j.cels.2023.12.004 38237552

[ref87] Wickham H. (2016). ggplot2: Elegant graphics for data analysis. Cham: Springer International Publishing.

[ref88] Worden A. Z. Follows M. J. Giovannoni S. J. Wilken S. Zimmerman A. E. Keeling P. J. (2015). Rethinking the marine carbon cycle: factoring in the multifarious lifestyles of microbes. Science 347:1257594. doi: 10.1126/science.1257594, PMID: 25678667

[ref89] Wu X. Liu Y. Weng Y. Li L. Lin S. (2022). Isolation, identification and toxicity of three strains of Heterocapsa (Dinophyceae) in a harmful event in Fujian, China. Harmful Algae 120:102355. doi: 10.1016/j.hal.2022.102355, PMID: 36470604

[ref90] Wu W. Lu H.-P. Sastri A. Yeh Y.-C. Gong G.-C. Chou W.-C.. (2018). Contrasting the relative importance of species sorting and dispersal limitation in shaping marine bacterial versus protist communities. ISME J. 12, 485–494. doi: 10.1038/ismej.2017.183, PMID: 29125596 PMC5776463

[ref91] Xie B. Huang J. Huang C. Wang Y. Shi S. Huang L. (2020). Stable isotopic signatures (δ13C and δ15N) of suspended particulate organic matter as indicators for fish cage culture pollution in Sansha Bay, China. Aquaculture 522:735081. doi: 10.1016/j.aquaculture.2020.735081

[ref92] Xie B. Huang C. Wang Y. Zhou X. Peng G. Tao Y.. (2021). Trophic gauntlet effects on fisheries recovery: a case study in Sansha Bay, China. Ecosyst. Health Sustain. 7:1965035. doi: 10.1080/20964129.2021.1965035

[ref93] Xu Q. Vandenkoornhuyse P. Li L. Guo J. Zhu C. Guo S.. (2022). Microbial generalists and specialists differently contribute to the community diversity in farmland soils. J. Adv. Res. 40, 17–27. doi: 10.1016/j.jare.2021.12.003, PMID: 36100325 PMC9481938

[ref94] Xue Y. Liu M. Chen H. Jeppesen E. Zhang H. Ren K.. (2022). Microbial hierarchical correlations and their contributions to carbon-nitrogen cycling following a reservoir cyanobacterial bloom. Ecol. Indic. 143:109401. doi: 10.1016/j.ecolind.2022.109401

[ref95] Yan Q. Liu Y. Hu A. Wan W. Zhang Z. Liu K. (2022). Distinct strategies of the habitat generalists and specialists in sediment of Tibetan lakes. Environ. Microbiol. 24, 4153–4166. doi: 10.1111/1462-2920.16044, PMID: 35590455

[ref96] Yang Y. Liu Q. Chai Z. Tang Y. (2015). Inhibition of marine coastal bloom-forming phytoplankton by commercially cultivated *Gracilaria lemaneiformis* (Rhodophyta). J. Appl. Phycol. 27, 2341–2352. doi: 10.1007/s10811-014-0486-0

[ref97] Yang J. W. Wu W. Chung C.-C. Chiang K.-P. Gong G.-C. Hsieh C. (2018). Predator and prey biodiversity relationship and its consequences on marine ecosystem functioning—interplay between nanoflagellates and bacterioplankton. ISME J. 12, 1532–1542. doi: 10.1038/s41396-018-0111-3, PMID: 29703955 PMC5956075

[ref98] Zhang J. (2016). Spaa: SPecies association analysis. 0.2.2. doi: 10.32614/CRAN.package.spaa

[ref99] Zhou W. Shen X. Xu Z. Yang Q. Jiao M. Li H.. (2024). Specialists regulate microbial network and community assembly in subtropical seagrass sediments under differing land use conditions. J. Environ. Manag. 370:122486. doi: 10.1016/j.jenvman.2024.122486, PMID: 39278015

[ref100] Zhu J. Ma Y. Huang L. Zhang W. (2022). Homogeneous selection is not always important in bacterial community in the eutrophic enclosed bay. Ecol. Process. 11:27. doi: 10.1186/s13717-022-00373-1

[ref101] Zhu W. Zhu M. Liu X. Xia J. Yin H. Li X. (2022). Different responses of bacteria and microeukaryote to assembly processes and co-occurrence pattern in the coastal upwelling. Microb. Ecol. 86, 174–186. doi: 10.1007/s00248-022-02093-7, PMID: 35927589

[ref102] Zinger L. Amaral-Zettler L. A. Fuhrman J. A. Horner-Devine M. C. Huse S. M. Welch D. B.. (2011). Global patterns of bacterial beta-diversity in seafloor and seawater ecosystems. PLoS One 6:e24570. doi: 10.1371/journal.pone.0024570, PMID: 21931760 PMC3169623

[ref103] Zubkov M. V. Sleigh M. A. Tarran G. A. Burkill P. H. Leakey R. J. G. (1998). Picoplanktonic community structure on an Atlantic transect from 50°N to 50°S. Deep Sea Res. Part I Oceanogr. Res. Pap. 45, 1339–1355. doi: 10.1016/S0967-0637(98)00015-6

