# Supplementary material for: Diversity and interdomain networks of bacterial, pico-protist and nano-protist communities in a marine ranching
Source: Front Microbiol. 2025 Jul 17;16:1620645. doi: 10.3389/fmicb.2025.1620645 (PMC12311637; doi:10.3389/fmicb.2025.1620645)
Supplement: Supplementary file 2 [file Supplementary_file_1.pdf]

Supplementary Material

1 Supplementary Figures and Tables

1.1 Supplementary Figures

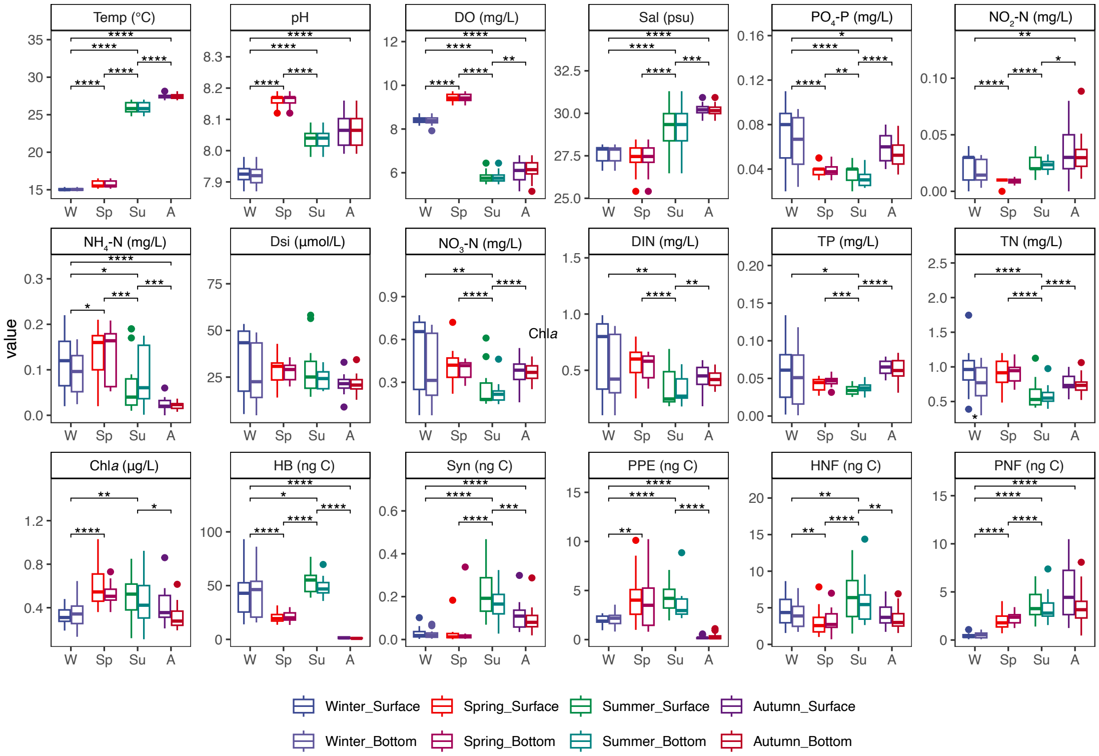

Supplementary Figure 1. Environmental factors at different seasons. Asterisk indicates significant differences at the level of $p < 0.05$ (*, $p < 0.05$; **, $p < 0.01$; ***, $p < 0.001$; ****, $p < 0.0001$). The abbreviations of seasons are: W, winter; Sp, spring; Su, summer; A, autumn. The abbreviations of environmental variables are: Temp, Temperature; DO, dissolved oxygen; Sal, salinity; PO4-P, phosphate; NO2-N, nitrite; NH4-N, ammonium; Dsi, silicate; NO3-N, nitrate; DIN, dissolved inorganic nitrogen; TN, total nitrogen; TP, total phosphorus; Chla, chlorophyll a; HB, heterotrophic bacteria; Syn, Synechococcus; PPE, photosynthetic picoeukaryotes; HNF, heterotrophic nano-sized flagellates; PNF, pigmented nano-sized flagellates.

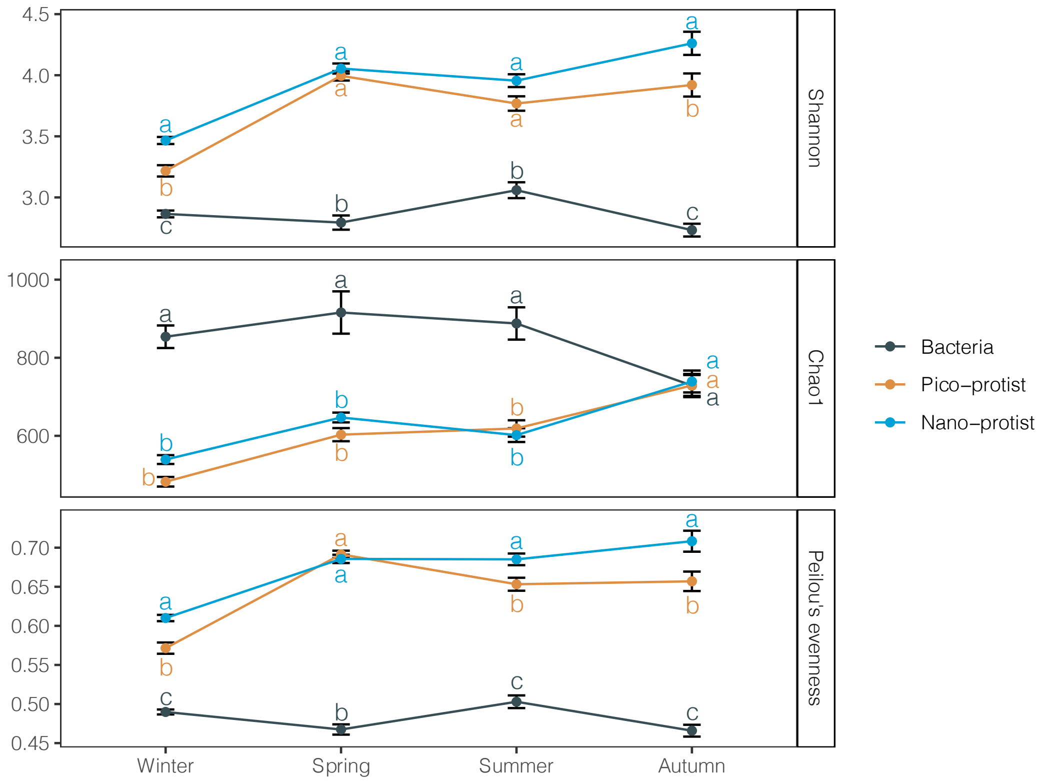

Supplementary Figure 2. Plots of changes in α-diversity indices of microbial communities during the four seasons. Multiple comparisons (LSD test) are calculated among taxa and seasons. Letters indicate significant differences ($p < 0.05$).

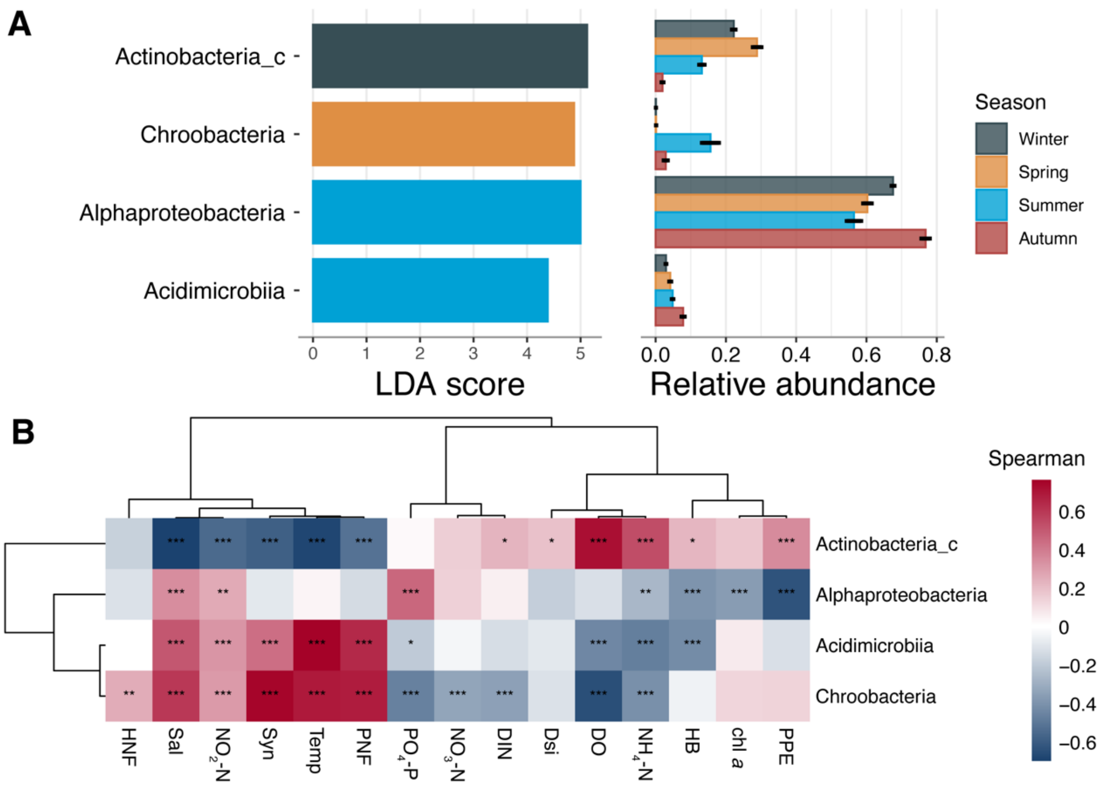

Supplementary Figure 3. (A) Bacterial taxa at class level with linear discriminant analysis (LDA) scores greater than 4.0 and their relative abundance over the four seasons. LDA scores were calculated using the linear discriminant analysis effect size (LEfSe) method. (B) Heatmap depicting Spearman's rank correlations between selected bacteria (LDA score > 4.0) and environmental factors. Environmental factors are abbreviated as described in Fig.S1.

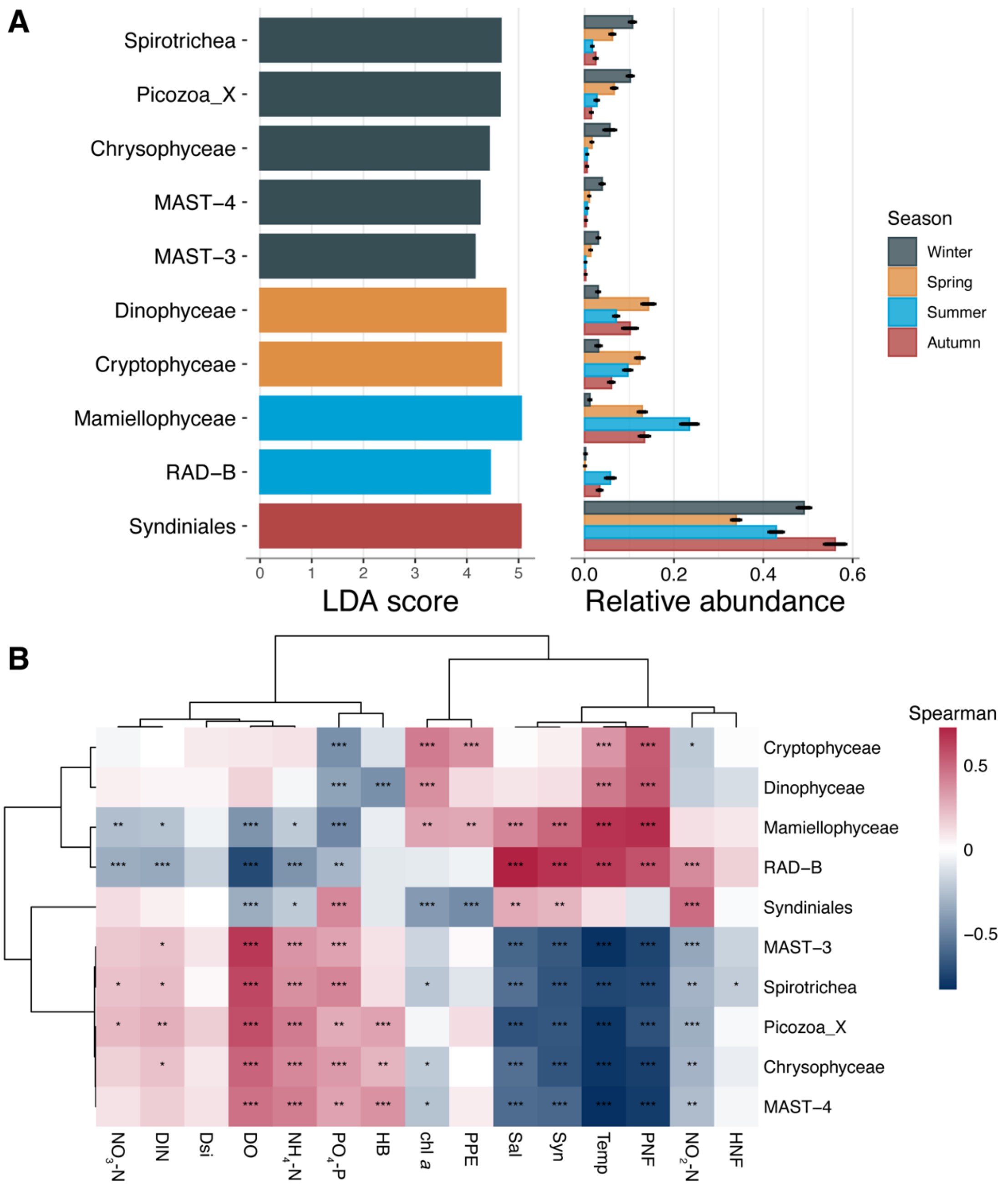

Supplementary Figure 4. (A) Pico-protist taxa at class level with LDA scores greater than 4.0 and their relative abundance over the four seasons. LDA scores were calculated using the LEfSe method. (B) Heatmap depicting Spearman's rank correlations between selected pico-protist (LDA score > 4.0) and environmental factors. Environmental factors are abbreviated as described in Fig.S1.

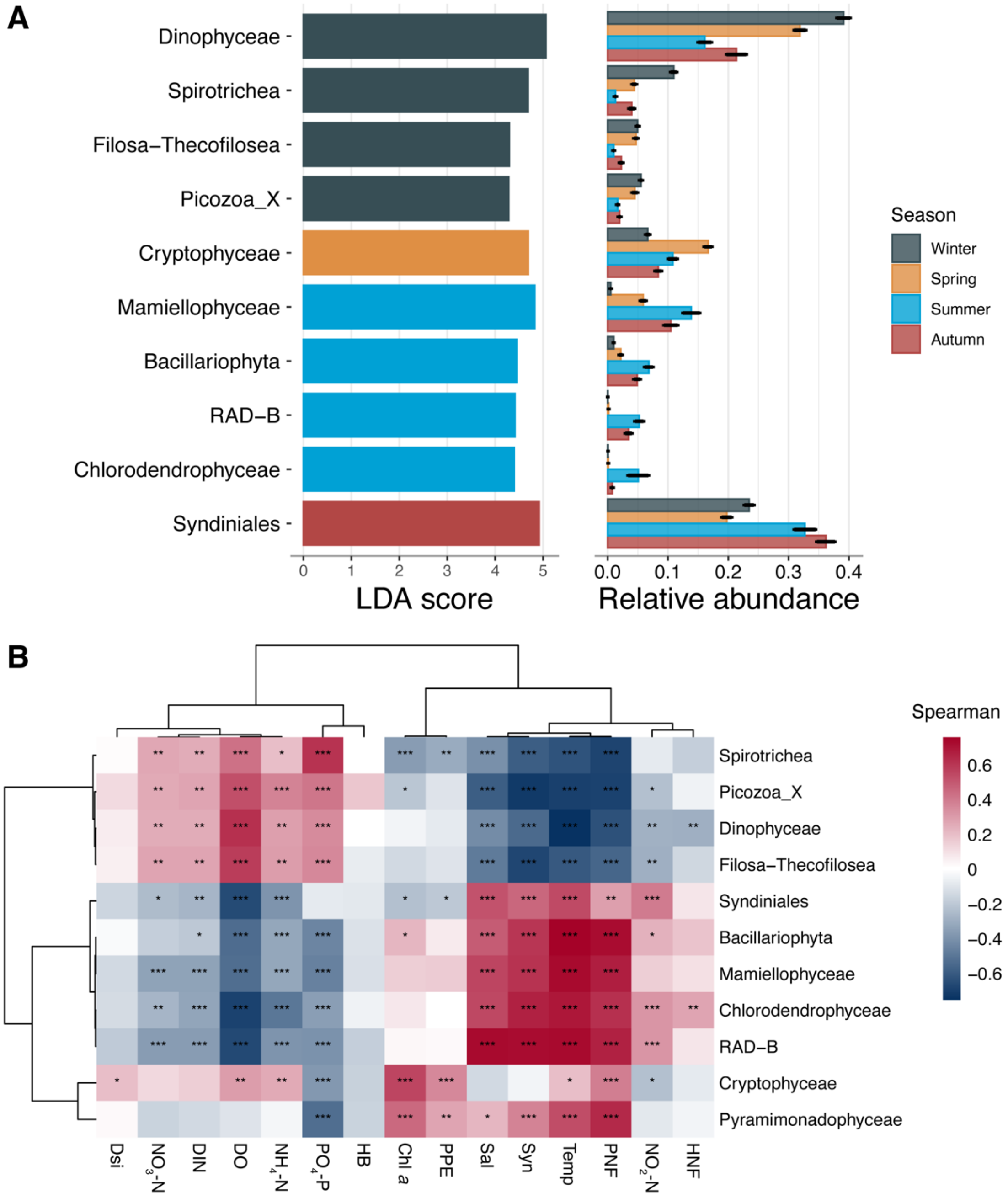

Supplementary Figure 5. (A) Nano-protist taxa at class level with LDA scores greater than 4.0 and their relative abundance over the four seasons. LDA scores were calculated using the LEfSe method. (B) Heatmap depicting Spearman's rank correlations between selected nano-protist (LDA score > 4.0) and environmental factors. Environmental factors are abbreviated as described in Fig.S1.

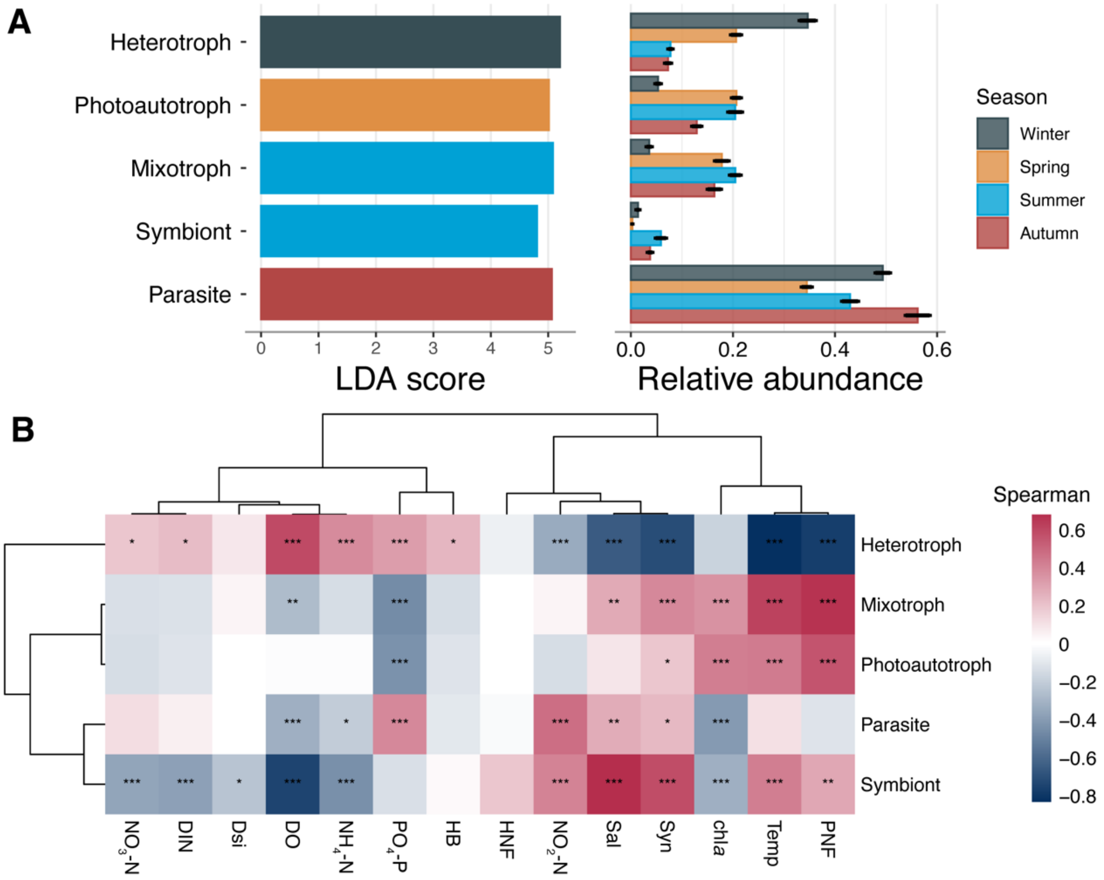

Supplementary Figure 6. Fig.S6 (A) Pico-protist taxa grouped by trophic modes with LDA scores greater than 4.0 and their relative abundance over the four seasons. LDA scores were calculated using the LEfSe method. (B) Heatmap depicting Spearman's rank correlations between selected pico-protist (LDA score > 4.0) and environmental factors. Environmental factors are abbreviated as described in Fig.S1.

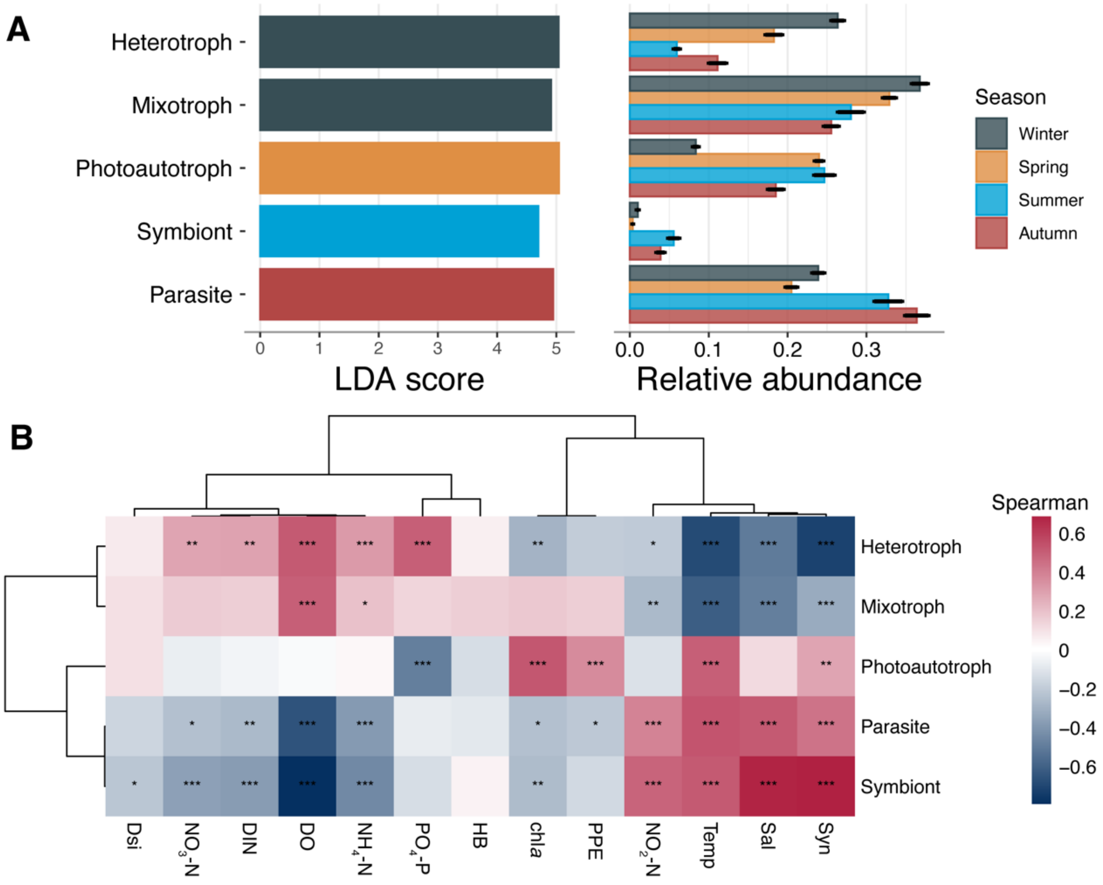

Supplementary Figure 7. (A) Nano-protist taxa grouped by trophic modes with LDA scores greater than 4.0 and their relative abundance over the four seasons. LDA scores were calculated using the LEfSe method. (B) Heatmap depicting Spearman's rank correlations between selected nano-protist (LDA score > 4.0) and environmental factors. Environmental factors are abbreviated as described in Fig.S1.

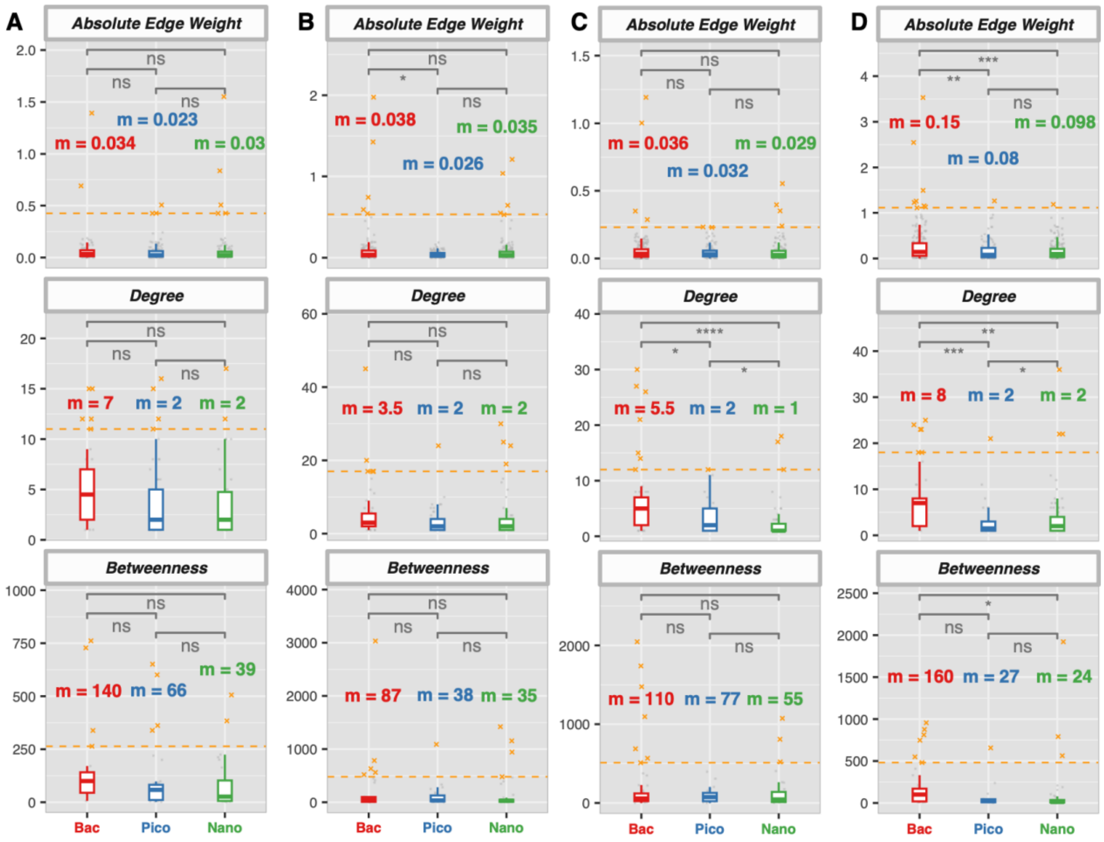

Supplementary Figure 8. Box plots of absolute edge weight, degree, and betweenness of its constituent bacteria (red), pico-ptotist (blue), and nano-protist (green) for (A) winter, (B) spring, (C) summer, and (D) autumn bacteria-pico-protists-nano-protists interdomain networks, with P values of mean differences calculated using Wilcoxon signed-rank test. The top 20th percentile of each factor presented as crosses and colored in orange. Significance level: ns, $p > 0.05$; *, $p < 0.05$; **, $p < 0.01$; ***, $p < 0.001$; ****, $p < 0.0001$.

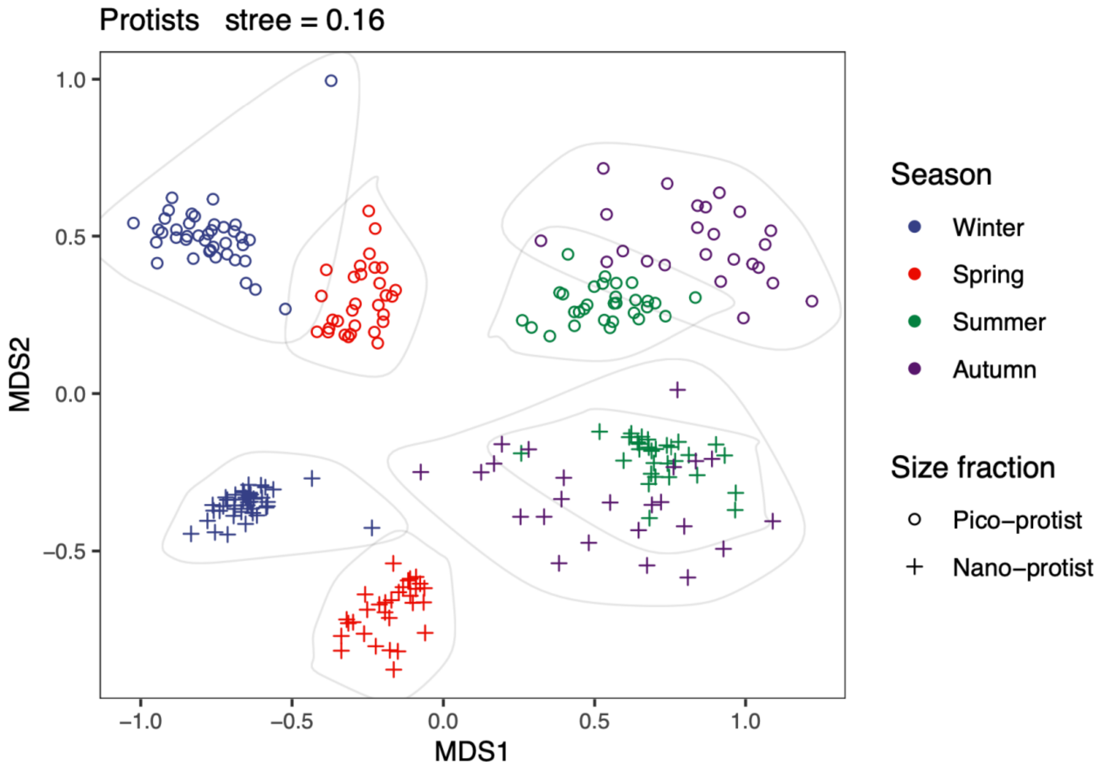

Supplementary Figure 9. Non-metric multidimensional scaling (NMDS) ordination of pico- and nano-protist communities in four seasons.

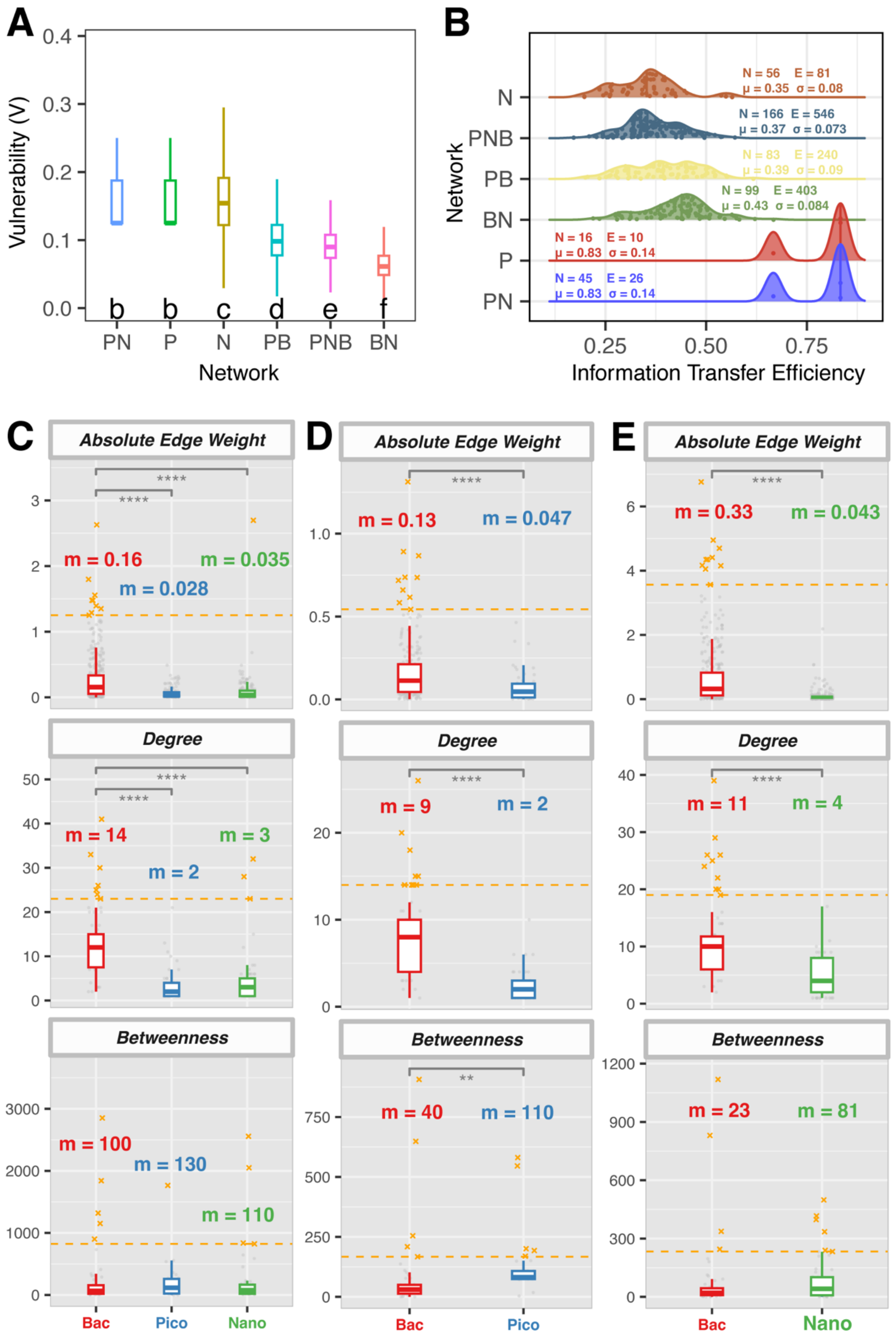

Supplementary Figure 10. Network analysis of robustness, information transfer efficiency and connectivity analysis of PNB, PB, BN, PN, P and N networks. (A) Vulnerability (V) inferred from

randomized attack robustness, and letters indicate significant differences ($p < 0.05$). (B) Network efficiency distribution curves in increasing order of average efficiency, with dots representing each node in the network. The text shows the number of nodes (N) and edges (E) of the network, as well as the mean (μ) and standard deviation (σ) of the curve. Box plots of absolute edge weight, degree, and betweenness of its constituent bacteria (red), pico-ptotist (blue), and nano-protist (green) for (C) PNB, (D) PB , and (E) BN networks, with P values of mean differences calculated using Wilcoxon signed-rank test. The top 20th percentile of each factor presented as crosses and colored in orange. Significance level: ns, $p > 0.05$; *, $p < 0.05$; **, $p < 0.01$; ***, $p < 0.001$; ****, $p < 0.0001$. Network name letter, B, bacteria; P, pico-protist; N, nano-protist.
